# A Dense Pyramidal Residual Network with a Tandem Spectral–Spatial Attention Mechanism for Hyperspectral Image Classification

**DOI:** 10.3390/s25061858

**Published:** 2025-03-17

**Authors:** Yunlan Guan, Zixuan Li, Nan Wang

**Affiliations:** 1 Key Laboratory of Mine Environmental Monitoring and Improving around Poyang Lake of Ministry of Natural Resources, East China University of Technology, Nanchang 330013, China; 2 School of Surveying and Geoinformation Engineering, East China University of Technology, Nanchang 330013, China; shmily@ecut.edu.cn (Z.L.); wangnan231426@163.com (N.W.); 3 Jiangxi Key Laboratory of Watershed Ecological Process and Information, East China University of Technology, Nanchang 330013, China

**Keywords:** hyperspectral image classification, convolutional neural network, tandem attention mechanism, dense pyramid residual network, residual unit, dilated convolution

## Abstract

In recent years, convolutional neural networks (CNNs) have become a potent tool for hyperspectral image classification (HSIC), where classification accuracy, computational cost, and generalization ability are the main focuses. In this study, a novel approach to hyperspectral image classification is proposed. A tandem spectral–spatial attention module (TAM) was designed to select significant spectral and spatial features automatically. At the same time, a dense pyramidal residual module (DPRM) with three residual units (RUs) was constructed, where feature maps exhibit linear growth; a dense connection structure was employed between each RU, and a TAM was embedded in each RU. Dilated convolution structures were used in the last two layers of the pyramid network, which can enhance the network’s perception of fine textures and features, improving information transfer efficiency. Tests on four public datasets, namely, the Pavia University, Salinas, TeaFarm, and WHU-Hi-HongHu datasets, were carried out, and the classification accuracies of our method were 99.60%, 99.95%, 99.81%, and 99.84%, respectively. Moreover, the method enhanced the processing speed, especially for large datasets such as WHU-Hi-HongHu. The training time and testing time of one epoch were 53 s and 1.28 s, respectively. Comparative experiments with five methods showed the correctness and high efficiency of our method.

## 1. Introduction

Hyperspectral remote sensing combines imaging technology with spectral technology to capture hyperspectral images (HSIs), which have tens or even hundreds of bands with rich spectral and spatial information [1]. Many intricate details that are difficult to obtain in wide-band multispectral images can be effectively detected in HSIs [2]. As a result, they have wide applications in fields such as ecological environmental protection [3], geological exploration [4], and agricultural production [5].

Hyperspectral image classification (HSIC) is the classification of each pixel of an HSI into a predetermined set of land-cover categories and the assignment of a unique land-cover category label [6]. This forms the foundation of hyperspectral remote sensing applications and has become a highly active research field in the hyperspectral community [7]. Due to problems such as high dimensionality, large data volumes, strong correlations among bands, limited training samples, inadequate feature extraction capabilities, and a variety of data description models, HSIC faces challenges. Features are the basis for HSIC. Earlier, classification features were extracted through manually designed algorithms [8,9,10,11,12,13,14,15,16]. With the development of artificial intelligence, deep learning technology, especially convolutional neural networks (CNNs), has been widely used in image processing. CNNs provide a more direct and powerful feature learning approach and can automatically learn complex feature representations from data without the need for manually designed feature extraction algorithms, thus reducing the reliance on manual feature design. Lecun et al. proposed LeNet-5 for handwritten number recognition [17], and this was the prototype of contemporary convolutional neural networks. The emergence of AlexNet [18] ushered in a new era for deep learning technology, making it a mainstream method for image classification and recognition [19,20,21]. Hu et al. [22] applied a 1D-CNN to extract spectral features, which provides valuable insights into the application of CNNs in HSIC. Cao et al. [23] introduced a 2D-CNN method based on active learning. This approach involves selecting pixels with the maximum information content for annotation, effectively improving accuracy while reducing costs. In such convolutional networks, each feature map in the previous layer requires corresponding convolutional kernel parameters for calculations, and thus, the number of convolutional kernels in the previous layer must match the number of channels in the feature map of the previous layer. However, hyperspectral images typically have hundreds of bands, which can potentially lead to an excessive number of convolutional kernels in a 2D-CNN, causing overfitting. Li et al. [24] first applied 3D convolutional neural networks (3D-CNNs) to the classification of hyperspectral imagery. They used hyperspectral image cubes as input without any preprocessing. The structure was solely composed of 3D-CNNs, which led to the significant loss of features for some small sizes or unevenly distributed land-cover types, and the computational cost was high. Li et al. [25] proposed a novel adaptive spatial–spectral feature learning (ASSFL) network to analyze spatial texture variations and learn robust adaptive features. CNNs and softmax normalization were applied to acquire shallow joint adaptive features, and then a stacked auto-encoder (SAE) was proposed to further extract deeper hierarchical features for the final classification. Paoletti et al. [26] proposed a 3D-CNN with a border-mirroring strategy to effectively process border areas in images, using graphics processing units (GPUs) to reduce the computation time and increase the accuracy of HSIC. Although 3D-CNNs can simultaneously utilize spectral and spatial information, they may be more complex than traditional 2D-CNNs, potentially requiring more parameters and computational resources. Zhong et al. [27] introduced a spectral–spatial residual network (SSRN) that combines 3D convolutional neural networks with the skip connection idea in residual networks to enhance the performance of hyperspectral image classification. The network is relatively shallow and has no effective feature propagation between convolutional layers, resulting in the underutilization of spectral and spatial features. Wang et al. [28] introduced a fast dense spectral–spatial convolution network (FDSSC) based on SSRN and DenseNet [29], which increased network depth while reducing training time and achieving better performance. However, the FDSSC framework uses 1 × 1 × d sized convolutional kernels to extract spectral features without taking into account the relationships between pixels and their neighbors. Researchers also pay attention to lightweight CNNs. Roy et al. [30] developed an end-to-end spectral–spatial squeeze–excitation (SE) residual bag-of-feature learning framework by constructing codebook representations of transformed features through exciting feature maps. This framework suppresses redundant feature maps and facilitates classification performance.

In CNNs, features are not treated equally in convolutional layers. To optimize feature extraction, attention mechanisms have been introduced to emphasize the importance of different features, enhancing useful bands or pixels and suppressing invalid information. Inspired by the convolutional block attention module (CBAM) [31], Ma et al. [32] proposed a double-branch multi-attention mechanism network (DBMA) for HSI classification. Based on the DBMA algorithm and an adaptive self-attention mechanism in a dual-attention network (DANet) [33], Li et al. [34] presented an end-to-end double-branch dual-attention mechanism network (DBDA) that utilized 3D convolutional layers and attention mechanisms for joint spatial–spectral feature extraction. Roy et al. [35] proposed an adaptive spectral–spatial kernel improved residual network that flexibly adjusts the size of the receptive field and utilizes an efficient feature recalibration mechanism to enhance classification performance. Xue et al. [36] proposed a hierarchical residual network with an attention mechanism to improve the performance of conventional networks. A hierarchical residual network was used to extract multiscale spatial and spectral features at a granular level, and an attention mechanism was used to set adaptive weights for spatial and spectral features of different scales. Chen et al. [37] constructed a deep CNN to extract spatial–spectral features and introduced a channel attention mechanism to recalibrate the extracted features. Wang et al. [38] proposed a global attention information interaction method for HSIC. Channel global attention features and spatial global attention features were extracted and then integrated through an information interaction module, making more comprehensive use of spectral and spatial information for classification. Yang et al. [39] proposed an end-to-end spectral–spatial attention bilateral network (SSABN). They first used a spectral–spatial attention module to enhance the useful bands or pixels; then, they designed a bilateral network with spatial and context paths to separately extract spatial and spectral features, which were finally fused. Kang et al. [40] proposed a spectral–spatial double-branch network (SSDBN) with an attention mechanism. The SSDBN was designed with two independent branches to extract spectral and spatial features, incorporating multiscale 2D convolution modules, long short-term memory, and an attention mechanism. Recently, state-space models (SSMs) have emerged as a promising alternative to traditional CNNs and Transformers for sequence modeling tasks. Among these, Mamba [41] introduces a selective state-space mechanism that dynamically adjusts model parameters based on input content, achieving linear computational complexity with respect to the sequence length. This makes Mamba particularly suitable for handling long-range dependencies in high-dimensional data, such as hyperspectral images. Based on this, MambaHSI [42] was proposed for hyperspectral image classification; it adapts the Mamba architecture by incorporating spectral–spatial selective mechanisms. MambaHSI effectively addresses challenges such as high dimensionality, data redundancy, and computational inefficiency, offering a lightweight yet powerful framework for feature extraction and classification. These advancements highlight the potential of SSMs in overcoming the limitations of traditional deep learning methods in HSIC.

According to a review of the existing research, most CNN-based methods for HSIC face three main challenges.

Redundancy in hyperspectral data: Hyperspectral data exhibit redundancy, with different bands and pixels contributing differently to network classification. End-to-end networks struggle to adaptively select useful bands while suppressing irrelevant ones. There is difficulty in highlighting pixels of the same class as the central pixel or useful pixels in the neighborhood of the central pixel and in suppressing pixels of different classes or irrelevant pixels.

Feature loss in deeper networks: As the depth of CNNs increases, feature loss becomes more severe. Subsequent layers cannot directly use features extracted from preceding layers, leading to issues such as gradient vanishing and overfitting.

Computational challenges in increasing the receptive field: Existing methods attempt to increase the receptive field by enlarging the size of convolutional kernels. However, when integrating information from a larger range in feature maps, the computational burden increases dramatically, potentially leading to memory overflow. This makes it hard to effectively capture correlations between different spectral bands and spatial relationships.

To address these issues and further improve the performance of HSIC, a novel method, named the dense pyramidal residual network with a tandem spectral–spatial attention mechanism (TAM-DPRN), is proposed. The key innovations of the method include the following.

Tandem attention module (TAM): A tandem attention module (TAM) that concatenates spectral and spatial attention mechanisms is constructed. By normalizing data in batches and inputting them into the network, the model first learns a set of attention weights in the spectral dimension to emphasize the most discriminative spectral features and then uses a spatial attention model to adaptively select spatial information. Thus, the most relevant spatial–spectral features are extracted for classification.

Dense pyramidal residual module (DPRM): A DPRM is constructed as the backbone network. The module increases the number of feature maps linearly in each residual unit (RU) and embeds TAMs hierarchically in each RU. It also introduces a reasonable dilation rate for the convolutional kernel in the last unit to increase the network width. The dense residual structure ensures that features extracted at each layer are faithfully mapped to subsequent layers, allowing for effective feature utilization and the extraction of more abstract features.

## 2. Methods

### 2.1. The Overall Framework of TAM-DPRN

**Figure 1:**
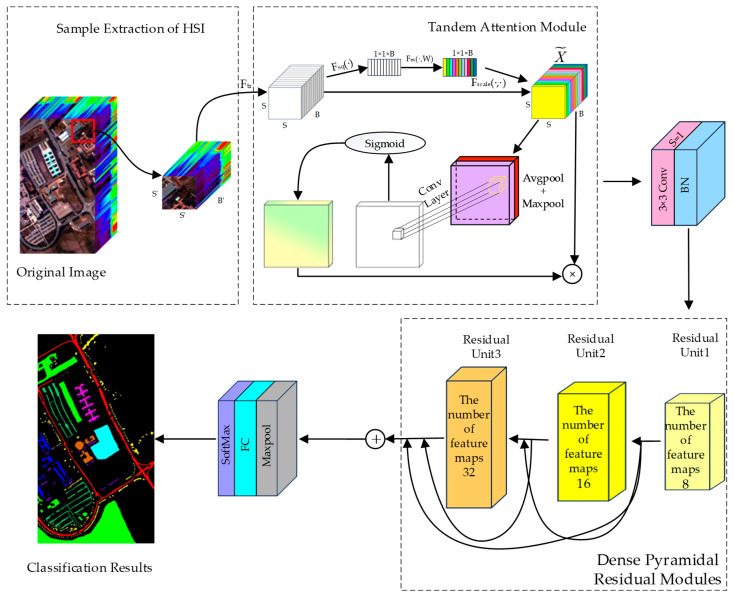
The network structure of TAM-DPRN.

The framework of TAM-DPRN is shown in Figure 1.

In order to make full use of spectral and spatial information, the network extracts pixel-centered blocks that contain all bands within a certain neighborhood around a pixel. These blocks are used as input for the CNN. The first layer of the model is the tandem attention module (TAM). By applying the TAM to input data, the model learns important spectral and spatial information. Next, the dense pyramidal residual module (DPRM) is designed to preserve the original spectral and spatial information using dense residuals. This design allows the network to be stacked to any depth. The use of residual learning and batch normalization (BN) [43] instead of the Dropout strategy [44] helps to avoid overfitting with limited training samples, thus improving classification accuracy. The TAM is embedded in an RU to enhance the learned final task features. Furthermore, dilated convolutions are employed in the last RU to learn the features of different scales. The model ensures that the feature map scale remains unchanged before and after convolution by padding all convolution operations. Each RU contains a global average pooling layer, a global max pooling layer, three convolutional layers, two batch normalization layers, eight activation function layers, and four linear layers. To reduce the number of parameters, global average pooling is used to aggregate spatial features learned from the last RU, facilitating efficient GPU operation. The aggregated features are then input into the softmax classifier for final classification. The entire network consists of 84 layers.

It is assumed that there is an HSI dataset $D\in R^{H\times W\times B}$, where *H* and *W* represent the height and width, and *B* is the number of spectral bands. The dataset includes N labeled pixels $X=\{x_{1},\;x_{2},\;\ldots ,\;x_{N}\}\in R^{1\times 1\times B}$, each associated with its corresponding one-hot encoded vector $Y=\{y_{1},\;y_{2},\;\ldots ,\;y_{N}\}\in R^{1\times 1\times K}$, where *K* represents the number of categories in the dataset. To adapt the data for the model’s input and extract spatial and spectral information comprehensively, the network creates pixel-centered blocks $Z=\{z_{1},\;z_{2},\;\ldots ,\;z_{N}\}\in R^{S^{\prime }\times S^{\prime }\times B}$ that include all spectral bands within a certain neighborhood around each pixel, where each cubic block $z_{i}$ is centered on a labeled pixel $x_{i}$. These three-dimensional patch blocks serve as input for the model to classify the corresponding central pixel $x_{i}$ in the HSI dataset.

Initially, the labeled data are randomly partitioned into training, validation, and testing sets, denoted as $Z_{\mathrm{train}}$, $Z_{\mathrm{val}}$, and $Z_{\mathrm{test}}$, respectively, with corresponding labels of $Y_{\mathrm{train}},\;Y_{\mathrm{val}}$, and $Y_{\mathrm{test}}$. Subsequently, model training and hyperparameter tuning are conducted; the model and hyperparameter settings proposed here use $Z_{\mathrm{train}}$ to optimize the model parameters. The trained model is then evaluated on $X_{\mathrm{val}}$ to find the hyperparameter combination that yields the best classification performance through cross-validation. Finally, the best-performing model is used on $Z_{\mathrm{test}}$ to evaluate its performance using the overall accuracy (OA), average accuracy (AA), and Kappa coefficient as evaluation metrics. The network follows a hybrid training–validation mode, where the best-trained model is applied to the HSI dataset, classifying all pixels to generate the final classification map.

### 2.2. Tandem Attention Module

The TAM consists of a spectral attention module and a spatial attention module. These two modules are connected in series. The TAM includes a global average pooling layer, a global max pooling layer, four linear layers, six activation function layers, and one convolutional layer. By applying the TAM to input data, the module firstly learns important spectral information and then spatial information. The TAM not only considers the adaptive adjustment and selection of spectral bands and spatial information but also addresses the need for further refinement of the learned spectral–spatial features. In the TAM, a soft attention mechanism is utilized to enhance the distinguishability of spectral features by learning a weighted vector that assigns weights to all features. The term “soft” in soft attention refers to the model assigning a probability distribution across the entire input sequence rather than focusing on a single fixed portion of the input. This enhances the model’s ability to capture long-range dependencies and relationships in the input data, enabling the model to adaptively identify spectral bands and information pixels that are crucial for HSIC.

Given an input feature map $d\in R^{S\times S\times B}$, spectral attention map $M_{\mathrm{se}}\in R^{1\times 1\times B}$, and spatial attention map $M_{\mathrm{sa}}\in R^{S\times S\times 1}$, the feature map *d* is derived from the pixel block *Z* through the convolutional transformation *F_tr_*, and $M_{\mathrm{se}}$ and $M_{\mathrm{sa}}$ are generated by selecting spectral bands in one dimension and spatial information in two dimensions, respectively. The calculation of these two types of attention is represented as follows, where ⨂ denotes element-wise multiplication.

(1) $$d^{\prime }=M_{\mathrm{se}}(d)⨂d$$

(2) $$d^{\prime \prime }=M_{\mathrm{sa}}(d^{\prime })⨂d^{\prime }$$

where $d^{\prime }$ represents the result after applying spectral attention weighting to the input feature map d, which enhances the focus on important spectral bands; $d^{\prime \prime }$ represents the result of further applying spatial attention weighting to $d^{\prime }$, which improves the ability to distinguish spatial information.

#### 2.2.1. Spectral Attention Module

**Figure 2:**
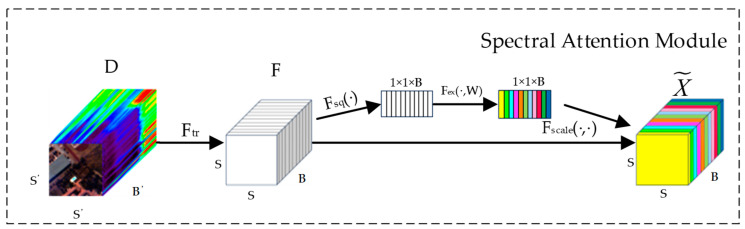
Spectral attention module.

The purpose of incorporating the spectral attention module is to effectively capture the correlations and importance between different bands when dealing with HSIs. The structure of the spectral attention module is illustrated in Figure 2.

The input features are mapped to a spectral weight vector through a function that represents the importance of each band. It is assumed that there is an input feature map $D\in R^{S^{\prime }\times S^{\prime }\times B^{\prime }}$, where the input feature map in this module consists of pixel blocks centered around each pixel, containing all of the bands within a certain neighborhood. Here, $S^{\prime }$ and $B^{\prime }$ represent the width and height of the pixel block and the total number of bands in the dataset. After a series of convolution operations $F_{\mathrm{tr}}$, the feature map $F\in R^{S\times S\times B}$ is obtained.

Global average pooling (GAP) calculates the average value of all pixels in the feature map, which aids in capturing the overall distribution and general features, thus providing a smoother feature representation. In section $F_{\mathrm{sq}}(\cdot )$, GAP is applied to each spatial position’s feature values, meaning that, for each channel *i*, its average is computed over the entire spatial extent, resulting in a B-dimensional channel-wise descriptor vector $z_{i}^{a}$, which is used to capture the overall features of each channel.

(3) $$z_{i}^{a}=\frac{1}{H\times W}\sum_{h=1}^{H}\sum_{w=1}^{W}x_{i,\;h,\;w}$$

where $z_{i}^{a}$ represents the overall feature information of channel *i*, $x_{i,\;h,\;w}$ represents the feature value of the *i*-th channel at the height *h* and width w in the input feature map, and *H* and *W* denote the height and width of the input feature map before average pooling, respectively.

In addition, each feature map also employs global max pooling (GMP) to emphasize the most significant features in an image. This helps capture locally important information with higher significance. Similar to global average pooling, the *i*-th channel of $z^{m}$ is expressed as follows:

(4) $$z_{i}^{m}=\max(x_{i,\;h,\;w})$$

where $x_{i,\;h,\;w}$ represents the feature value of the *i*-th channel at the height h and width w in the input feature map and $z_{i}^{m}$ represents max feature value of *i*-th channel.

In section $F_{\mathrm{ex}}(\cdot ,W)$, a small neural network is designed to learn the importance of each channel, aiming to fully capture the interactions and dependencies related to the bands. To limit model complexity and aid generalization, this network consists of two fully connected (FC) layers and two activation functions. The gating mechanism is parameterized using two FC layers: a dimensionality-reduction layer with parameters $W_{1}$ and a reduction ratio of *r*, followed by a ReLU activation function and then a dimensionality-increasing layer with parameter $W_{2}$. Through this section, an excitation vector representing the weight for each channel can be obtained. The calculation of the excitation vector is shown as follows:

(5) $$s_{i}=F_{\mathrm{ex}}(z,\;W)=\sigma (g(z,\;W))=\sigma (W_{2}\delta (W_{1}z))$$

where $W_{1}\in R^{\frac{B}{r}\times B}$, $W_{2}\in R^{B\times \frac{B}{r}}$, δ represents the ReLU activation function, σ is the sigmoid activation function, and $s_{i}$ denotes the weights of channel *i*. Variable *z* represents the channel-wise statistical information after GMP. To reduce the number of parameters, the network parameters for the GAP and GMP operations are shared by two fully connected layers, and the outputs of these two pooling operations are then summed. Finally, the computed weight $s_{i}$ is multiplied by the corresponding feature value $x_{i,\;h,\;w}\in R^{h\times w\times 1}$ in the original feature map of the respective channel, resulting in a scaled feature map. This operation is denoted as follows:

(6) $${\tilde{X}}_{i,\;h,\;w}=s_{i}\cdot x_{i,\;h,\;w}$$

where ${\tilde{X}}_{i,\;h,\;w}$ is the feature value of the *i*-th channel at the height *h* and width w, and it is obtained through a scaling operation. It is the original feature value, and $x_{i,\;h,\;w}$ is the result obtained by scaling based on the importance of the channel. Through this scaling operation, the feature map is adjusted according to the importance of each channel, enhancing information from important channels while suppressing less crucial channel information. In the end, all scaled channel feature maps are recombined by $F_{\mathrm{scale}}(\cdot ,\cdot )$ to obtain an enhanced feature map.

The spectral attention module automatically learns the weights for each spectral band through the above process, in the output feature map $\tilde{X}$, different colors indicate the varying weights obtained through computation for each band in different layers. This enhances the network’s ability to represent features at the spectral level, aiding the network in better capturing crucial information. It adaptively selects useful spectral bands for classification.

#### 2.2.2. Spatial Attention Module

**Figure 3:**
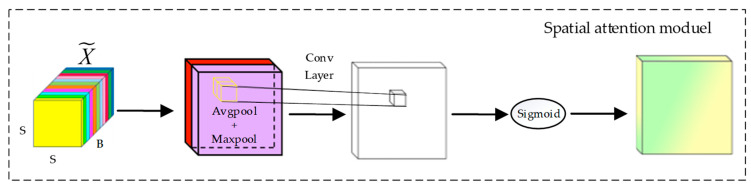
Spatial attention module.

The inclusion of the spatial attention module is intended to effectively utilize the spatial relationships between pixels and enhance the extraction of relevant features from hyperspectral data, thereby improving the accuracy and robustness of the classification model. Figure 3 illustrates the structure of the spatial attention module.

In this figure, $\tilde{X}$ represents the output of the previous section’s Spectral Attention Module and serves as input to the Spatial Attention Module. As with the spectral attention module, this section applies GAP and GMP on the input over all spectral bands. These two pooling operations capture the average and maximum values of all channels in the spatial dimension, generating a spatial average feature map $Z_{h,\;w}^{a}\in R^{h\times w\times 1}$ and a spatial max feature map $Z_{h,\;w}^{m}\in R^{h\times w\times 1}$. This step can be expressed as follows:

(7) $$Z_{h,\;w}^{a}=\frac{1}{B}\sum_{b=1}^{B}{\tilde{X}}^{\prime }(h,\;w)$$

(8) $$Z_{h,\;w}^{m}=\max({\tilde{X}}^{\prime })$$

where ${\tilde{X}}^{\prime }$ is the value of the *b*-th channel at position (*h*, *w*).

Subsequently, the outputs of these two pooling operations are concatenated along the spectral dimension, forming a new feature map $m\in R^{h\times w\times 2}$. The red and purple colors represent the feature map weights of $\tilde{X}$ after passing through GAP and GMP, respectively. By incorporating information from both average and max pooling, spatial features can be better captured. Next, the new feature map is processed with a two-dimensional convolution layer with padding and a sigmoid activation function. This restricts the output values to the range of 0 to 1, resulting in the final spatial attention weight map $A\in R^{h\times w\times 1}$.

(9) $$A=\sigma ([Z_{h,\;w}^{a},\;Z_{h,\;w}^{m}]*f^{3\times 3})$$

where * is the convolution operation, σ represents the sigmoid function, and $f^{3\times 3}$ represents the convolution operation with a filter size of 3 × 3.

This weight map is then used to adjust the spatial features of the original hyperspectral data. In the TAM, the spatial attention module’s final output is obtained by applying spatial multiplication using the final attention weight map A to the input features of the spatial attention module ${\tilde{X}}_{i,\;h,\;w}$.

### 2.3. Construction of the Dense Pyramidal Residual Module (DPRM)

Given that ResNet can effectively address the issues of vanishing gradients and insufficient expressiveness in training deep networks [45], a residual network structure is introduced into the backbone network. This involves overlaying an identity mapping of the original hyperspectral input data with the feature maps extracted after feature extraction. This approach fully exploits the correlations between hyperspectral data. Simultaneously, the TAM is embedded into the network to enhance the learning of hierarchical features relevant to the final classification task.

**Figure 4:**
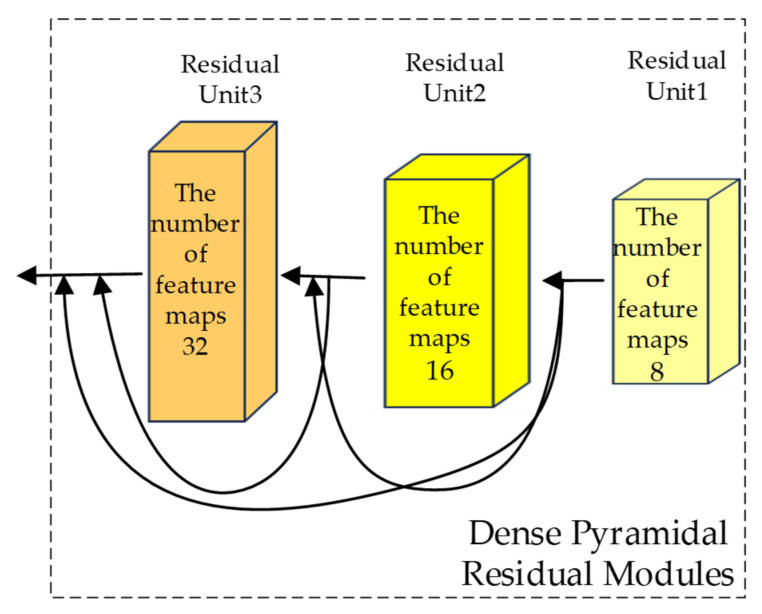
Dense pyramidal residual modules.

The DPRM proposed in this study is composed of three stacked RUs, and multiple-scale dense connections are employed between each RU to concurrently extract feature information at different scales. The input of the *n*-th RU is composed of the outputs of all preceding blocks and the input of the first RU. The output of this RU will serve as the input for the next RU, as illustrated in Figure 4. Unlike traditional methods that rely on fixed-size convolutional kernels or separate branches for multiscale feature extraction, the DPRM employs a pyramid-like structure with dilated convolutions in the final residual unit (RU). This design allows the network to progressively expand its receptive field while maintaining spatial resolution, capturing both fine-grained details and broader contextual information.

**Figure 5:**
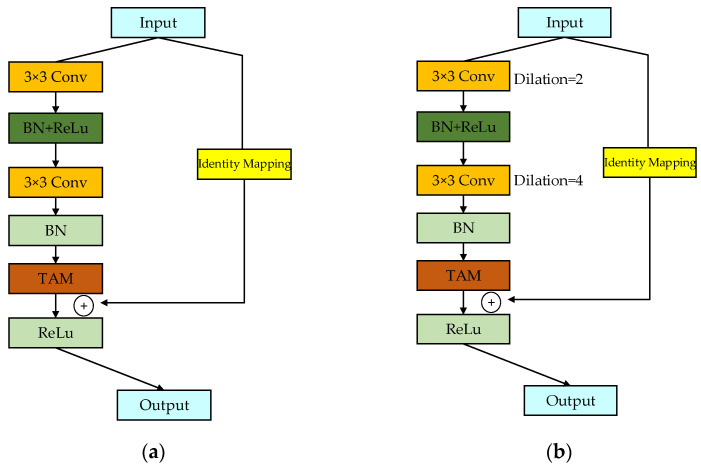
The structure of a residual unit (RU): (**a**) the structure of RU1 and RU2; (**b**) the structure of RU3.

Each RU consists of two convolutional layers, two BN layers, a TAM, two ReLU activation functions, and a residual connection. The structure of each RU is depicted in Figure 5. The input data are scaled to an appropriate range through the nonlinear activation function of the BN layer, followed by an enhancement in the neural network’s expressive capability using the nonlinear ReLU activation function. The equation for the BN layer is defined as follows:

(10) $${\mathrm{bn}}_{i}=\gamma \frac{x_{i}-\mu_{B}}{\sqrt{\sigma_{B}^{2}+\epsilon }}+\beta$$

(11) $$\mu_{B}=\frac{1}{m}\sum_{i=1}^{m}x_{i}\;$$

(12) $$\sigma_{B}^{2}=\frac{1}{m}\sum_{i=1}^{m}{\left(x_{i}-\mu_{B}\right)}^{2}$$

where $x=(x_{1},x_{2},\ldots ,x_{m})$ denotes input data and *m* represents the number of samples in a batch. $\mu_{B}$ stands for the mean of the batch input data, and $\sigma_{B}^{2}$ represents the variance in the batch data. The parameters γ and β are learnable scaling and shifting factors used to restore the network’s representational capacity. ϵ is a small constant introduced to prevent the denominator in the normalization process from being zero.

The dense connections between RUs ensure that features extracted at each layer are fully utilized in subsequent layers. This contrasts with methods such as FDSSC and DBMA, which may suffer from feature loss or redundancy due to their independent branch designs.

The BN layer effectively mitigates internal covariate shifts, maintaining the stability of the data distribution, and readjusts the normalized output using the two learnable parameters. The output from the final ReLU function is then fed into a convolutional layer to extract more informative features. In this study, the addition of pixel values along the channels is achieved by combining the RUs.

Let $m^{i}$ represent the input features at the i-th layer. Within each RU, there are a total of six hidden layers. $w^{i+1}$ and $b^{i+1}$ denote the weights and biases of the neurons at the (i+1)-th layer. Therefore, we have Equations (13)–(16):

(13) $$m^{i+2}=\mathrm{ReLU}(\mathrm{BN}(m^{i}\times w^{i+1}+b^{i+1}))$$

(14) $$m^{i+4}=\mathrm{BN}(m^{i+2}\times w^{i+3}+b^{i+3})$$

(15) $$m^{i+5}=m^{i+4}\cdot F_{\mathrm{TAM}}(m^{i+4})$$

(16) $$m^{i+6}=\mathrm{ReLU}(m^{i}+m^{i+5})$$

where $F_{\mathrm{TAM}}(m^{i+4})$ refers to using the output of the fourth layer as the input for the fifth TAM layer. This is processed with both a spectral attention module and a spatial attention module, resulting in the output feature map $m^{i+5}$. *ReLU*(·), *BN*(·), and $F_{\mathrm{TAM}}(\cdot )$ denote the ReLU activation function, batch normalization, and TAM operation, respectively.

In RU1 and RU2, the convolution kernel size is 3, the stride is 1, the padding is 1, and the dilation rate is 1. In RU3, building on this foundation, the dilation rates of the two convolutional layers are set to 2 and 4, respectively, with the corresponding padding also being set to 2 and 4. Overlaying these two dilated convolutions allows the network to have a larger receptive field with the same parameters and computational cost, enabling the synthesis of information at multiple scales. This enhances the network’s understanding and classification capabilities for complex images. Setting a too-large dilation rate might lead to a lack of correlations between feature maps from different layers, resulting in the loss of local image information and making the model less sensitive to image details. The design of this network ensures that the feature map size remains unchanged before and after convolution. Additionally, the number of output feature maps for each RU increases linearly. As the network deepens, the model covers more positions, retaining more detailed information at different layers while balancing the workload among all modules. This reduces the time complexity of the network.

Each output block from the 3D-CNN with the attention mechanism is closely connected to the subsequent 2D-CNN layer. Subsequently, the outputs obtained from all three blocks are fused through concatenation. In the final layer, the concatenation of each feature map uses a 3 × 3 max pooling and a fully connected layer. Finally, softmax is applied to obtain scores for each category from the refined feature maps. This network design incrementally increases the diversity of higher-order spectral–spatial properties across layers, thus enhancing the performance of the network based on HSI data. The use of 2D convolutions and a linear growth in feature maps reduces the computational burden compared with that of 3D-CNN-based methods, making the DPRM more suitable for large-scale datasets such as WHU-Hi-HongHu.

### 2.4. Loss Function and Optimization

An appropriate loss function and optimizer are needed to optimize the proposed model. Cross-entropy loss is commonly used in classification tasks, as it measures a model’s predictive performance by comparing the output probability distribution of the model with the true label’s probability distribution. The loss function for cross-entropy is given as follows:

(17) $${\mathrm{Loss}}_{\mathrm{CE}}=-\frac{1}{N}\sum_{i=1}^{N}\sum_{c=1}^{C}y_{i,\;c}\log(p_{i,\;c})$$

where *N* is the number of samples. *C* is the number of classes. $y_{i,c}$ is a true label indicating whether the *i*-th sample belongs to the *c*-th class. If the *i*-th sample belongs to the *c*-th class, $y_{i,c}$ = 1; otherwise, $y_{i,c}$ = 0. $p_{i,c}$ is the probability predicted by the model that the *i*-th sample belongs to the *c*-th class.

Given the large number of samples in our dataset and their relatively dispersed distribution, we conducted experiments using mini-batches. The challenge with mini batches is the need to update weights by dividing by different gradients each time. Consequently, we utilized the RMSprop optimizer, which maintains a moving average of the squared gradients for each weight and then divides the gradient by the root mean square. Mathematically, it can be expressed as follows:

(18) $${E[g^{2}]}_{t}=\beta {E[g^{2}]}_{t-1}+(1-\beta ){\left(\frac{\delta C}{\delta w}\right)}^{2}$$

(19) $$w_{t}=w_{t-1}-\frac{\eta }{\sqrt{{E[g^{2}]}_{t}}}\frac{\delta C}{\delta w}$$

where ${E[g^{2}]}_{t}$ represents the moving average of the gradient squares, $\frac{\delta C}{\delta W}$ denotes the gradient of the loss function with respect to the weights, η stands for the learning rate, and β signifies the moving average parameter. From the equation, it can be observed that by dividing by the square root of the gradient, the learning rate is adjusted. However, since we only have the current batch’s gradient estimates, we need to use its moving average.

## 3. Materials and Experiments

### 3.1. HSI Datasets

To validate the effectiveness and universality of the proposed method, four publicly available HSI datasets were selected for experimental verification.

The UP dataset [46] was acquired using the German Reflective Optics Spectral Imaging System (ROSIS-03) over Pavia University in northern Italy. It consists of nine land-use types. The spatial resolution is 1.3 m, and the spectral range varies from 0.43 to 0.86 μm. It contains a total of 115 bands with a spectral resolution of 4 nm, but only 103 bands not polluted by noise were used in this study. Table 1 presents the ground object class and sample numbers for the UP dataset.

The SA dataset [46] was collected using the Airborne Visible InfraRed Imaging Spectrometer (AVIRIS) over Salinas Valley in California, USA, with a 3.7 m spatial resolution. The spectral range of the images is from 0.4 to 2.5 μm, with a total of 224 bands. Like the UP dataset, 20 bands with strong water vapor absorption were excluded (including bands 108–112, 154–167, and 224). The remaining 204 bands were used for the classification experiments. The SA dataset includes 16 land-cover types, such as fallow, celery, and vineyards. These images were available only as at-sensor radiance data. Table 2 presents the ground object class and sample numbers for the SA dataset.

Table 3 presents the ground object class and sample numbers for the TeaFarm dataset [47]. The TeaFarm dataset was collected using the Pushbrom Hyperspectral Imager (PHI) over a tea plantation in Fanglu Village, Changzhou City, Jiangsu Province, China. The spectral range of the images is 417–855 nm, with 80 spectral bands. The spatial resolution is 2.25 m, and the geographical coordinates of the image center are 119°22′53″ E, 31°40′39″ N. This consists of 348 × 512 pixels.

**Figure 6:**
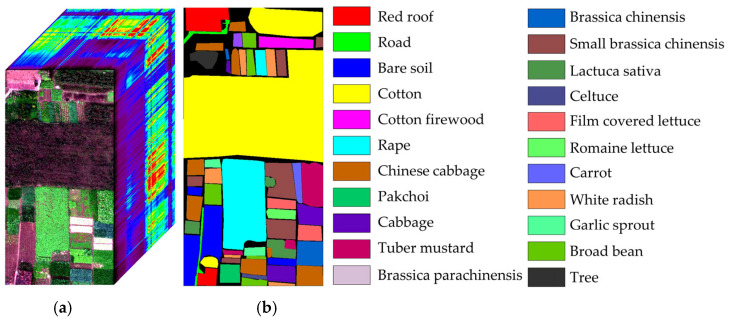
HongHu dataset. (**a**) Pseudo-color map; (**b**) ground-truth image.

The WHU-Hi-HongHu dataset [48,49] contains data from Honghu City, Hubei Province, China. The data were collected with a 17 mm focal length Headwall Nano-Hyperspec imaging sensor mounted on a DJI Matrice 600 Pro unmanned aerial vehicle (UAV). Compared with the other datasets, the area is a complex agricultural scene with many classes of crops, and different cultivars of the same crop are also planted, including Chinese cabbage, cabbage, Brassica chinensis, and small Brassica chinensis. In total, there are 22 land-cover classes in the target collection area. The UAV flew at an altitude of 100 m with a sensor capable of capturing data across 270 distinct spectral bands within the range of 400 to 1000 nm. The spatial resolution of the images is 0.043 m. The pseudo-color image and corresponding ground-truth labels are illustrated in Figure 6.

### 3.2. Experimental Configuration

All experiments in this study were conducted on a system equipped with a 13th Gen Intel(R) Core(TM) i5-13490F @ 2.50 GHz processor, 32 GB of memory, and an NVIDIA GeForce RTX 3060 Ti GPU with 8GB of VRAM. The system ran Windows 10 Professional. The software used for the experiments was PyTorch 2.0.1, a deep learning framework based on CUDA 12.2 and Python 3.8.16.

To fully exploit the model’s performance, the experimental settings were as follows. Random proportions of samples were selected from the datasets for training, testing, and validation. During training and validation, the data were randomly shuffled to enhance the model’s generalization ability. To enhance computational efficiency and make the most of spectral information, the original data were normalized. Subsequently, image patches centered on a pixel and encompassing all original spectral bands were extracted to serve as inputs for the model. The spectral attention mechanism within the model adaptively selected bands that were most conducive to feature representation and classification.

Prior to starting the training of the network, the weights and biases of all convolutional neural networks (CNNs) were initialized using Kaiming initialization [50]. The network underwent 200 epochs of training using the RMSprop optimizer [51] with a momentum of 0.9. The weight decay was set to 0.0001, which served as a regularization technique by adding a penalty term to the loss function to control the complexity of the model. In the experiment, a weight decay value of 0.0001 was set to restrain large weights during optimization, controlling the model’s complexity and helping to prevent overfitting.

Table 4 provides details of the hyperparameter settings for the different datasets. These hyperparameters were determined through experimentation, and the specific details can be seen in Section 3.3.

### 3.3. Setting of Hyperparameters

This section primarily focuses on discussing the impact of hyperparameters on the model’s classification results in order to determine the optimal hyperparameters for each dataset. The hyperparameters include the batch size, sample spatial neighborhood size, optimizer selection, dataset partitioning ratio, learning rate, and weight decay. We used the control variable method across all experiments.

#### 3.3.1. Impact of the Batch Size on the Classification Results

**Figure 7:**
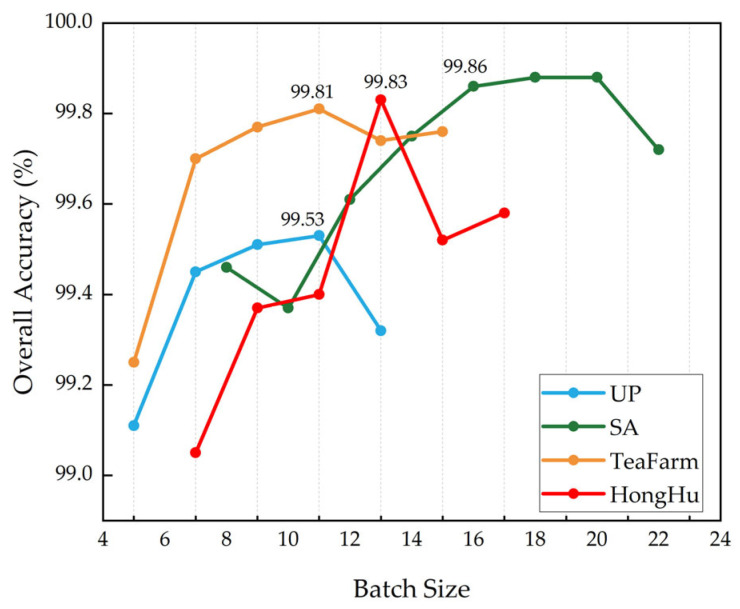
Accuracy changes with different batch sizes for the datasets.

The batch size refers to the number of data samples that are input into the model simultaneously for parameter updates during each iteration. In HSIC tasks, the dimensionality is high while the sample quantity is relatively small. Choosing a smaller batch size not only helps reduce the model’s dependence on specific data within the batch but also alleviates the computational burden during each parameter update. Different batch sizes were experimented with for the UP, SA, TeaFarm, and HongHu datasets, using the sets {5, 7, 9, 11, 13}, {8, 10, 12, 14, 16, 18, 20, 22}, {5, 7, 9, 11, 13, 15}, and {7, 9, 11, 13, 15, 17}, respectively, as shown in Figure 7.

As the batch size increases, the OA metrics for the four datasets exhibit a trend of initially rising and then declining. For the UP and TeaFarm datasets, a batch size of 11 yields the highest classification accuracy. For the SA and HongHu datasets, the optimal batch sizes are 16 and 13, respectively. Therefore, considering the dataset size and model complexity, the selected batch sizes for the UP, SA, TeaFarm, and HongHu datasets are 11, 16, 11, and 13, respectively.

#### 3.3.2. Impact of the Sample Spatial Neighborhood Size on the Classification Results

The sample spatial neighborhood size represents the size of the patch centered around a pixel containing all original image bands, and this is input into the network. This hyperparameter determines the spatial information range around a pixel that will be used for classification to some extent. Different HSIs exhibit distinct feature distributions, and choosing an appropriate spatial neighborhood size is crucial for the model to achieve optimal classification results while maintaining computational efficiency.

**Figure 8:**
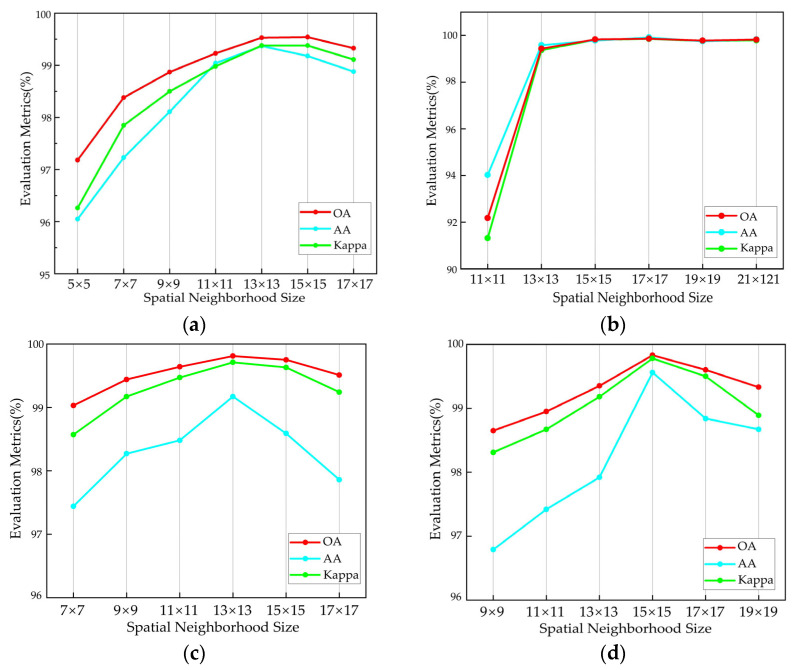
Effects of different spatial neighborhood sizes on the classification results. (**a**) UP dataset; (**b**) SA dataset; (**c**) TeaFarm dataset; (**d**) HongHu dataset.

In this study, with other parameters held constant, different spatial neighborhood sizes were experimented with. The results are illustrated in Figure 8. It is shown that, apart from the SA dataset, the three metrics for the other three datasets all exhibit an initial increase, reaching a peak before decreasing as the spatial neighborhood size increases. For the UP and TeaFarm datasets, when the optimal neighborhood size is set to 13 × 13, all metrics achieve their highest values. This trend occurs because the two datasets share similarities in the sizes and distributions of different types, with both containing a mix of various small and large types that are unevenly distributed.

For the SA dataset, the classification accuracy shows a rapid increase initially and then stabilizes after the neighborhood size reaches 15 × 15. This is because the SA dataset contains many larger land-cover types, and similar types tend to cluster together with relatively regular shapes and uniform distributions. Therefore, when the neighborhood size reaches a certain dimension, the network can effectively leverage the contextual information of each land-cover type. This contributes to a smoother classification result and stable accuracy. Although there is no significant change in the overall accuracy and Kappa coefficient for the SA dataset at neighborhood sizes of 15 × 15, 17 × 17, and 19 × 19, the 17 × 17 neighborhood size demonstrates excellent average accuracy. So, for the SA dataset, the optimal neighborhood size is set to 17 × 17.

For the HongHu dataset, the overall distribution of land-cover types is similar to that in the SA dataset, with regular patterns; however, irregular distributions exist in some small categories, such as roads, pak choi, etc. Additionally, this dataset includes various varieties of the same crop, such as Chinese cabbage, pak choi, celtuce, lactuca sativa, etc. Therefore, when tuning parameters for this dataset, the accuracy change curve is not as stable as that of the SA dataset, and the optimal values for the three metrics are reached when the neighborhood size is 15 × 15.

#### 3.3.3. Impact of the Dataset Partitioning Ratio on the Classification Results

Properly partitioning the training, validation, and test sets enables the model to learn from experience during the training phase through gradient descent, continuously reducing the training error. In the validation phase, different models and hyperparameter combinations were evaluated to find the optimal model parameters. This helps the model achieve better generalization on the test set. During the test phase, the performance of the model obtained from the validation phase was assessed on a new dataset to evaluate its generalization ability, ultimately determining the model’s quality.

For the four datasets, classification experiments were conducted using the partitioning ratios of {1:1:8, 1.5:1:7.5, 2:1:7, 2.5:1:6.5, 3:1:6, 3.5:1:5.5, 4:1:5}. The classification accuracy is presented in Table 5.

For the UP dataset, the model performs best when using a partition ratio of 1.5:1:7.5. As the size of the training set increases and the size of the test set decreases, the model’s performance starts to decline due to overfitting. This occurs because redundancy among the training samples leads to confusion in the model. When many training samples have similar spectra, they do not contribute additional beneficial information, which can cause the network to perform poorly, reflecting a decrease in performance.

The TeaFarm dataset shares similarities with the UP dataset, but it has a higher abundance of certain land-cover types (such as tea plants and sweet potatoes) with small and scattered distribution areas. Therefore, it is necessary to moderately increase the ratio between the training and test sets. This adjustment allows the network to learn the unique features of different categories more comprehensively under similar distribution conditions, thereby improving classification performance. For the SA and HongHu datasets, the model achieves optimal performance with a partition ratio of 3:1:6. In this case, the model has enough training samples to learn the features of regularly distributed data.

Taking all of the metrics into account, the best partition ratio for the UP dataset is 1.5:1:7.5, that for the TeaFarm dataset is 2:1:7, and that for the SA and HongHu datasets is 3:1:6. These partition ratios can balance the number of training samples, validate model performance, and ensure testing generalization performance, ensuring that the model achieves optimal performance in both training and testing.

#### 3.3.4. Impact of the Learning Rate on the Classification Results

**Figure 9:**
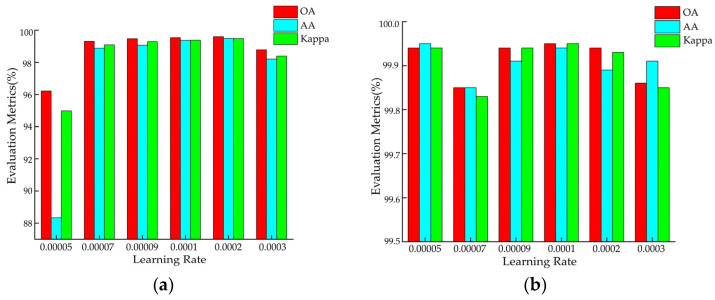

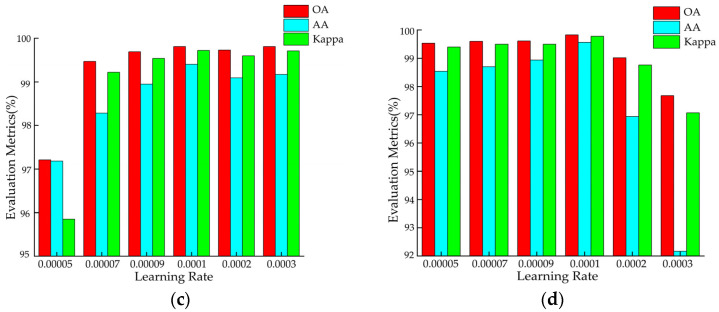
Effect of the learning rate on the classification results. (**a**) UP dataset; (**b**) SA dataset; (**c**) TeaFarm dataset; (**d**) HongHu dataset.

In deep neural networks, the learning rate is a crucial hyperparameter that determines the magnitude of each parameter update. It controls the step size of gradient descent and, thus, plays a key role in the optimization process. In this study, we employed the grid search method to find the optimal learning rate for the proposed model on each dataset. The considered learning rate set is {0.00005, 0.00007, 0.00009, 0.0001, 0.0002, 0.0003}. The classification results are shown in Figure 9. Based on the experimental results, a learning rate of 0.0002 was selected for the UP dataset, while a learning rate of 0.0001 was chosen for the other datasets. By systematically testing different learning rates, unnecessary experiments and training time can be reduced.

#### 3.3.5. Impact of Optimizer Selection on the Classification Results

In the optimizer comparison experiment, four mainstream adaptive optimizers—RMSprop, Adam, AdamW, and Nadam—were selected for a horizontal comparison to investigate the impacts of different gradient update mechanisms on model performance. Among them, Adam achieves fast convergence by integrating momentum gradients with adaptive learning rates (RMSprop), making it suitable for scenarios with a non-stationary target. AdamW decouples weight decay from parameter updates based on Adam, alleviating the issue of decreased generalization ability caused by the coupling of adaptive learning rates and regularization, particularly for complex tasks. Nadam introduces Nesterov momentum to enhance the foresight of gradient direction, improving convergence stability while optimizing the parameter update path. The experimental design employs the control variable method, keeping all parameters constant except for the optimizer parameters, with overall accuracy as the evaluation metric. The results are shown in Table 6.

According to the results of the experimental comparison, RMSprop achieved overall classification accuracies of 99.60%, 99.95%, 99.81%, and 99.84% on the PaviaU, Salinas, TeaFarm, and HongHu datasets, respectively, outperforming the Adam-series optimizers. This is primarily attributed to the gradient second-order moment exponential decay mechanism of RMSprop, which adaptively adjusts the learning rate by calculating the root mean square of the gradients within a window. This mechanism prevents over-updating sensitive bands due to a global learning rate, making it particularly suitable for handling the non-stationary feature distributions commonly found in hyperspectral data. Additionally, the gradient-smoothing mechanism of RMSprop effectively suppresses the impact of transient gradient fluctuations caused by the noise commonly present in hyperspectral image data. In contrast, while the Adam-series optimizers accelerate convergence by introducing a momentum mechanism, their first-order moment estimation tends to excessively smooth the gradient direction, leading to directional bias in regions of spectral feature abruptness. Although Nadam’s Nesterov momentum accelerates convergence, it is more susceptible to interference from local minima. Therefore, RMSprop, with its parameter independence, dynamic balancing capability, and computational efficiency, was identified as the optimal choice for hyperspectral image classification tasks.

#### 3.3.6. Impact of Weight Decay on the Classification Results

**Figure 10:**
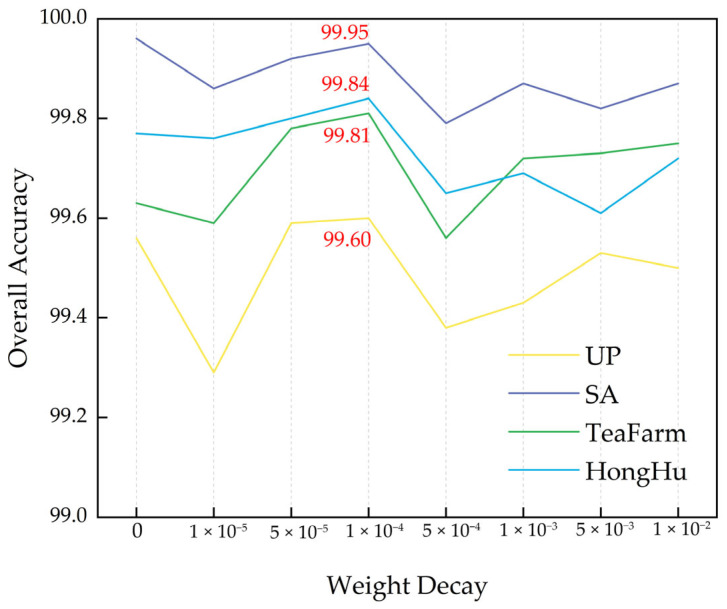
Effect of the learning rate on the classification results.

In the hyperparameter selection experiments on the model, weight decay, as a crucial regularization technique, was employed to balance the model’s complexity and generalization capability by constraining the weight norm, and it was identified as a key regulatory factor in preventing overfitting. To investigate the impacts of different weight decay values on model performance, a set of decay values of [0, 1 × 10^−5^, 5 × 10^−5^, 1 × 10^−4^, 5 × 10^−4^, 1 × 10^−3^, 5 × 10^−3^, and 1 × 10^−2^] was selected for comparative experiments. These chosen decay values spanned a range from no decay to substantial decay, aiming to evaluate how the model performance varied with different regularization strengths, thereby providing a theoretical basis for the selection of the weight decay hyperparameter. The subsequent experiments were designed to comprehensively demonstrate the effects of different weight decay values on classification performance, and the results are shown in Figure 10.

The experimental results demonstrated that a non-monotonic relationship between weight decay values and classification accuracy was observed across the four hyperspectral datasets. When the weight decay value was set to 1e−4, peak performance was achieved in the PaviaU (99.60%), Salinas (99.95%), TeaFarm (99.81%), and HongHu (99.84%) datasets, which was proven to be a direct manifestation of balanced regularization effects in hyperspectral image classification. This phenomenon was attributed to the dual regulatory nature of weight decay in controlling model capacity, where insufficient regularization (values < 1 × 10^−4^) led to models being prone to overfitting with spectral noise from redundant bands, whereas excessive regularization (values > 1 × 10^−4^) resulted in the learning of discriminative features from subtle spectral variations being suppressed. The 1 × 10^−4^ value was found to optimally balance this trade-off, a characteristic that proved particularly critical for hyperspectral data characterized by high-dimensional feature spaces and class-specific spectral variations. At this specific value, moderate L2-norm constraints were implemented, enabling simultaneous suppression of noise interference and preservation of sensitivity to critical spectral–spatial features. This mechanism effectively reconciled model complexity with generalization capabilities, demonstrating exceptional adaptability to the characteristic distributions of hyperspectral imaging data. Additionally, in the SA dataset, where crop planting patterns exhibited regular spatial arrangements, the joint spectral–spatial features were more readily extracted by the moderately regularized model, ultimately achieving a peak OA of 99.84%.

### 3.4. Methods for Comparison

To verify the performance and efficiency of our method, six methods were selected for comparison. To demonstrate the advantages of deep learning methods over traditional machine learning approaches, we chose a typical machine learning algorithm: SVM [8]. Inspired by the residual structure, we selected ResNet34 [45]. Additionally, we selected the FDSSC [28], DBMA [32], and DBDA [34] methods because they are authoritative methods published in recent years, and their overall ideas and modules are relatively similar to those in this study. We also selected the MambaHSI [42] method for comparison because it is a state-of-the-art method. We can analyze the advantages of our method based on subtle structural differences and explain the rationality of our method. These advanced deep learning models are all based on 3D-CNN methods, and they treat target pixels and adjacent pixels as inputs. In the experiments, all methods adopted a train–validate-as-you-go approach.

For the SVM algorithm, we chose the radial basis function (RBF) as the kernel function, and, prior to classification, PCA was utilized to reduce the dataset’s dimensionality. We used the cumulative explained variance ratio to determine the optimal number of principal components. This could decrease computational complexity while preserving as much original information as possible. Specifically, for the UP dataset, the first four principal components were retained, with a gamma kernel coefficient of 0.1 and a penalty coefficient C of 100. For the SA dataset, the first three principal components were retained, with a gamma kernel coefficient of 0.1 and a penalty coefficient C of 20. For the TeaFarm dataset, the first eight principal components were retained, with a gamma kernel coefficient of 0.1 and a penalty coefficient C of 100. Lastly, for the HongHu dataset, the first six principal components were retained, with an ‘auto’ gamma kernel coefficient (meaning the inverse of the number of sample features) and a penalty coefficient C of 1. Additionally, each classifier used the patch size specified in the original papers.

This experimental design allows for a fair comparison of TAM-DPRN with various methods. Moreover, by incorporating techniques such as PCA dimensionality reduction, traditional machine learning methods may become more competitive when handling high-dimensional data. This comprehensive evaluation can provide insights into the performance of different methods.

## 4. Results and Discussions

### 4.1. Performance Analysis

#### 4.1.1. Results and Analysis of the UP Dataset

**Figure 11:**
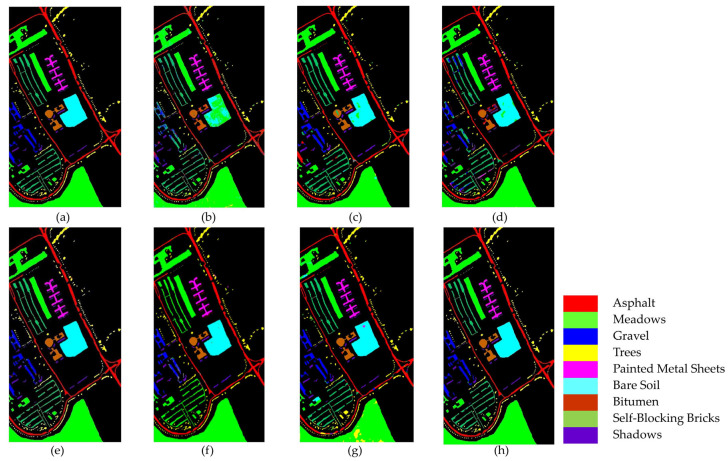
Classification maps of different methods on the UP dataset. (**a**) Ground truth; (**b**) SVM; (**c**) FDSSC; (**d**) DBMA; (**e**) DBDA; (**f**) ResNet34; (**g**) MambaHSI; (**h**) TAM-DPRN.

Figure 11 and Table 7 present the classification results of different methods on the UP dataset.

In Figure 11, it is clear that the SVM, FDSSC, and DBMA methods have noticeable classification errors in the Bare Soil category. In addition, the MambaHSI method was not only found to misclassify the Meadows category at the lower edge of the image but also incorrectly classified a portion of the Bare Soil as Painted Metal Sheets. The DBMA, DBDA, and ResNet34 methods display noise within an area of Gravel and a small number of Bricks. The misclassification issue is more severe in SVM, where much salt-and-pepper noise occurs.

As evident in Table 7, our TAM-DPRN method achieves the highest OA, AA, and Kappa coefficients, which are 99.60%, 99.49%, and 99.48%, respectively. Apart from the category of Gravel, the overall classification accuracy for the other categories is also above 99%. Due to the limited training samples and the lack of spatial information for classification, SVM has the lowest classification accuracy. FDSSC, which incorporates a dense connection structure in the network, outperforms ResNet34 by improving the classification accuracy of Bare Soil, Gravel, and Bricks by 0.49%, 1.34%, and 4.57%, respectively. Although DBMA, which is based on FDSSC, extracts spatial and spectral features through two independent branches and introduces an attention mechanism, it tends to overfit the training data when the training samples are extremely scarce, resulting in a 5.83% decrease in OA. MambaHSI, which is based on a state-space model, demonstrates potential in modeling the long spectral sequences of hyperspectral data, particularly in categories dominated by spectral features, such as Painted Metal Sheets and Meadows, where it exhibits robust performance. The Kappa coefficient (98.75%) surpasses that of most methods used for comparison. However, its solely spectral-oriented design leads to insufficient utilization of spatial information, resulting in significantly lower accuracy in space-sensitive categories, such as Gravel and Bricks, as well as in categories with scarce training samples, such as Bare Soil and Shadows, compared with TAM-DPRN. Moreover, the lack of explicit regularization strategies exposes it to the risk of overfitting in small-sample scenarios, limiting its overall performance with an OA of 94.70% and an AA of 96.21%. Despite the UP dataset lacking labeled samples and having a smaller data dimension in categories such as Metal Sheets, Asphalt, and Shadows, TAM-DPRN extracts spatial and spectral features from two independent attention mechanism branches and, thus, ensures that the model does not overfit the training data even when training samples are scarce. On the other hand, with DBDA adding residual connections to DBMA, the increased depth of the model makes gradient descent more challenging. In such cases, the network may focus more on unimportant details and overlook genuinely important features. DBDA exhibits a decrease in classification accuracy of 2.21%, 0.74%, and 1.34% for these three categories compared with DBMA. TAM-DPRN achieves stable and reliable performance under conditions of limited data by fully utilizing adaptive attention mechanisms at each layer, selecting appropriate loss functions according to the data characteristics, and employing an early stopping strategy within 50 epochs to prevent overfitting. Although TAM-DPRN exhibits slightly lower classification accuracy for certain categories compared with the other methods, the difference is not significant, indicating a favorable overall performance. Compared with the other five methods, our proposed approach achieves an overall accuracy improvement of 15.52%, 0.74%, 6.57%, 1.04%, and 1.39%, respectively.

The comprehensive analysis of Table 7 and Figure 11 indicates that for the UP dataset, TAM-DPRN effectively learns features from various categories, capturing their distinct characteristics, reducing both omission and commission errors, and, thereby, achieving the best classification accuracy.

#### 4.1.2. Results and Analysis of the SA Dataset

**Figure 12:**
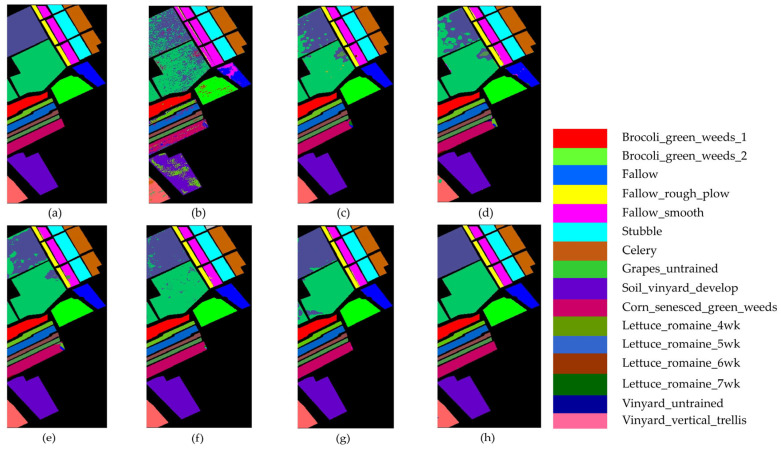
Classification maps of different methods on the SA dataset. (**a**) Ground truth; (**b**) SVM; (**c**) FDSSC; (**d**) DBMA; (**e**) DBDA; (**f**) ResNet34; (**g**) MambaHSI; (**h**) TAM-DPRN.

Table 8 and Figure 12 illustrate the comparative classification results on the SA dataset. From Table 8, it can be observed that the DBMA model shows a significant improvement over the FDSSC model due to the larger sample size and higher dimensionality of the SA dataset compared with the UP dataset. As a result, the classification accuracy increases by 1.66%. Although both DBDA and FDSSC do not show significant improvements in overall accuracy (OA), the classification result images indicate that DBDA exhibits stronger spatial continuity. Notably, the MambaHSI method achieves competitive performance, with 98.41% OA and 99.27% AA, particularly excelling in complex classes (e.g., 96.94% in Vinyard_untrained categories). Its design leverages a state-space model architecture with bidirectional scanning mechanisms, allowing it to dynamically capture long-range spectral dependencies while maintaining computational efficiency. Unlike traditional CNNs, MambaHSI adaptively weights spectral bands through its selective retention mechanism, filtering redundant information and enhancing discriminative features. However, its spatial modeling capabilities may be constrained compared with those of TAM-DPRN, as evidenced by the lower OA (98.41% vs. 99.95%). This gap suggests that while MambaHSI effectively models spectral sequences, the fusion of multiscale spatial–spectral features in TAM-DPRN provides more comprehensive contextual awareness. The TAM-DPRN method achieves accuracy metrics exceeding 99.9% across all three accuracy indicators. This is because TAM-DPRN allows the computer to adaptively identify the spectral bands that are beneficial for classification, emphasizing pixels of the same class as the central pixel or useful pixels within the pixel neighborhood. It also enables the fusion and superposition of feature maps across different layers. Consequently, its performance is significantly superior to that of the other five methods, with overall accuracy improvements of 10.17%, 4.91%, 3.25%, 4.14%, and 1.33%, respectively. The average accuracy and the Kappa coefficient also improve to varying degrees. These results indicate that TAM-DPRN excels in HSIC tasks, especially in integrating spectral and spatial information and in adaptive band selection. Additionally, the model successfully classifies all 10 categories, showcasing its superior performance.

As shown in Figure 12, SVM, FDSSC, DBMA, DBDA, and MambaHSI perform poorly in this task, showing considerable noise and spots in the results. While ResNet34’s overall performance is close to that of TAM-DPRN, it contains more internal noise. MambaHSI achieves joint spatial–spectral modeling through a bidirectional scanning mechanism, preserving the ability to capture global features. As a result, it demonstrates superior sensitivity to subtle spectral differences compared with ResNet34, showing better performance in the classification of the Fallow_rough_plow and Corn_senesced_green_weeds categories. It is evident that the TAM-DPRN method has a significant advantage. This is attributed to TAM-DPRN’s reasonable design of two dilated convolutions in the last RU, which broadens the receptive field and maintains the continuity of spatial information, thereby reducing noise pollution in the receptive field.

#### 4.1.3. Results and Analysis of the TeaFarm Dataset

**Figure 13:**
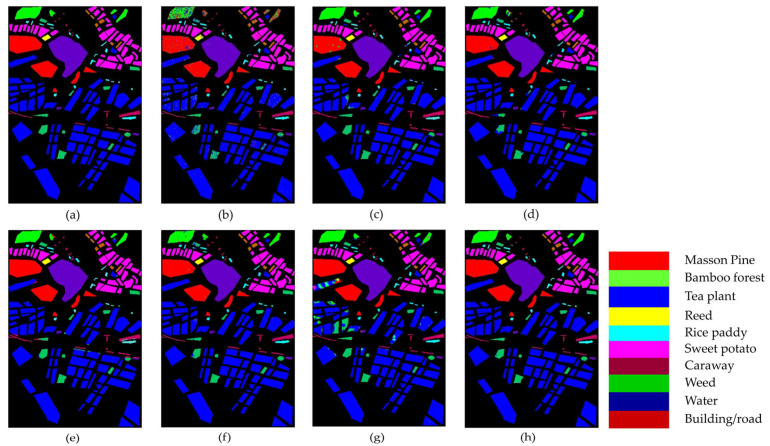
Classification maps on the TeaFarm dataset. (**a**) Ground truth; (**b**) SVM; (**c**) FDSSC; (**d**) DBMA; (**e**) DBDA; (**f**) ResNet34; (**g**) MambaHSI; (**h**) TAM-DPRN.

The classification results for the TeaFarm dataset are provided in Table 9 and Figure 13. For the classification of Reeds and Rice Paddies, TAM-DPRN achieves an accuracy of 100%, and for the Tea plant category, which has the largest number of samples in the dataset, the model’s classification accuracy significantly surpasses that of the other models. Compared with SVM, FDSSC, DBMA, DBDA, and ResNet34, the overall accuracy of our method improves by 14.97%, 0.68%, 0.8%, 0.67%, and 0.62%, respectively. Although the average accuracy is slightly lower than that of DBMA, the Kappa coefficient shows a substantial increase. Notably, MambaHSI exhibits a unique performance pattern; despite its relatively low OA (96.12%), it achieves a perfect Kappa coefficient (100%) and high AA (98.48%). This contradiction suggests that MambaHSI excels in balancing inter-class confusion (especially for minority classes, such as Reeds, with 100% accuracy) but struggles with dominant categories (e.g., 93.36% for Tea plants). Its state-space architecture with bidirectional spectral scanning enables the adaptive modeling of global spectral correlations, which likely reduces misclassification between similar spectral profiles. However, the lack of explicit spatial constraints (unlike TAM-DPRN’s pixel neighborhood weighting) may lead to fragmented predictions in homogeneous regions, explaining its lower OA. Additionally, its computational efficiency from linear-time complexity allows lightweight deployment but sacrifices fine-grained spatial feature fusion. Based on the table, the TAM-DPRN method achieves an OA of 99.81%, an AA of 99.40%, and a Kappa coefficient of 0.9972. In different datasets, due to the insufficient number of training samples, the classification results of DBMA exhibit an unstable trend. However, for the TeaFarm dataset, considering the classification accuracy of the categories with fewer samples, such as Reeds and Caraway, both SVM and ResNet34 perform poorly.

Figure 13 clearly illustrates the classification performance of the seven methods. The performance of SVM, FDSSC, DBMA, and MambaHSI is relatively poor, as indicated by large patches of noise and speckles in the classification maps. ResNet34 shows suboptimal performance in classifying small distributed areas of Weeds and has minor misclassification within Bamboo forests. FDSSC and DBMA exhibit significant misclassification issues within objects. Overall, DBDA performs slightly better than the aforementioned methods, but it struggles with edge information processing, leading to misclassification at the edges of Bamboo forests, Weed, Buildings/roads, and some Tea plants. MambaHSI misclassifies the Tea plant category as Weeds in areas with small, patchy distributions. TAM-DPRN effectively addresses the poor edge classification problem by appropriately setting the dilation factor in the dilated convolution. Compared with the other models, TAM-DPRN produces classification results with less edge noise and fewer speckles on the TeaFarm dataset, demonstrating superior performance.

#### 4.1.4. Results and Analysis of the HongHu Dataset

**Figure 14:**
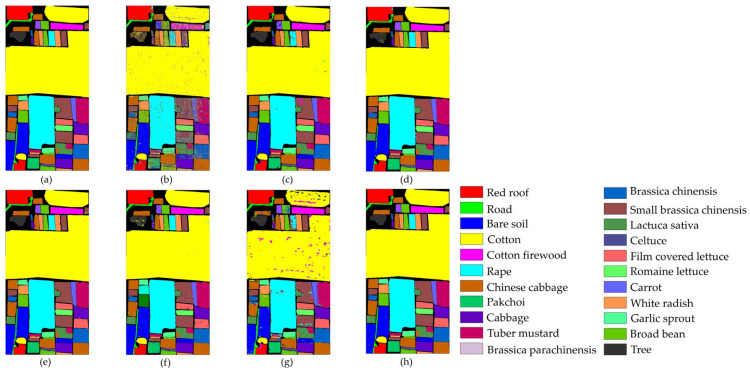
Classification maps on the HongHu dataset. (**a**) Ground truth; (**b**) SVM; (**c**) FDSSC; (**d**) DBMA; (**e**) DBDA; (**f**) ResNet34; (**g**) MambaHSI; (**h**) TAM-DPRN.

Table 10 and Figure 14 present the classification results of the various methods on the HongHu dataset, aiming to validate the performance of the TAM-DPRN method in handling large-scale datasets.

As shown in Table 10, the three indicators of the OA, AA, and Kappa coefficient are all optimal with the TAM-DPRN method. Compared with the other five methods, the overall classification accuracy increased by 15.23%, 1.05%, 0.75%, 1%, and 1.63%, respectively. For Rape, Cabbage, and Romaine Lettuce, TAM-DPRN can accurately classify them without errors. Despite these crops belonging to the same type, their spatial distribution and morphological characteristics can differ significantly due to variations in growing environments and growth stages. Consequently, difficult-to-classify crops, such as Chinese cabbage, pak choi, and tuber mustard, show significantly improved accuracy. In contrast, MambaHSI exhibits a mixed performance; while it achieves perfect accuracy in specific challenging categories such as celtuce and Broad Bean, its overall accuracy lags behind that of TAM-DPRN. For different varieties of the same crop, such as Film-Covered Lettuce and Romaine Lettuce, the classification performance is also superior to that of other methods. Overall, the accuracy of the TAM-DPRN method in each category exceeds 99%, indicating that the method can capture distinct features between different classes. Compared with methods that independently extract spectral and spatial information, such as FDSSC, DBMA, and DBDA, the spatial attention module in TAM-DPRN is more effective due to its adaptive spectral-weight-based joint feature fusion mechanism. The spectral–spatial features obtained by TAM-DPRN are more abstract and comprehensive, enabling the classification of some regions that are difficult to distinguish accurately.

In Figure 14, it is evident that the SVM, FDSSC, ResNet34, and MambaHSI methods produce significant internal noise for this dataset. The performance of both DBMA and DBDA is relatively good; however, their ability to classify Bare Soil and roads is suboptimal. Additionally, they tend to produce a significant amount of edge misclassification, as observed in the classification maps for Broad Bean and Romaine Lettuce. However, MambaHSI‘s limited spatial modeling capability (lacking explicit spatial attention modules such as those of TAM-DPRN) leads to inconsistencies in homogeneous regions, such as Chinese cabbage and White Radish, but this phenomenon is particularly obvious in the Cotton and Cotton Firewood Interior categories, where spatial texture variations are critical. Furthermore, MambaHSI’s reliance on sequential spectral processing, while computationally efficient, causes it to struggle to fuse multiscale spatial–spectral features hierarchically, resulting in fragmented predictions for morphologically similar crops (e.g., cabbage vs. Chinese cabbage). TAM-DPRN shows a highly accurate and smooth classification map. Particularly at the boundaries between different classes, the generated map is exceptionally precise.

For datasets such as the HongHu dataset, where the land-cover types are complex and difficult to classify without relying on spectral information, TAM-DPRN’s classification map shows reduced noise and spots, demonstrating superior overall performance. This indicates the model’s superior robustness and feature extraction capability in the HSIC task.

By comparing the experimental results on the four datasets, it is shown that the proposed method TAM-DPRN demonstrates excellent classification performance across all four datasets. It not only achieves the highest classification accuracy but also exhibits the least noise and spots in the classification result images, achieving good classification results in edge details.

The effectiveness of the proposed method in achieving favorable classification results is primarily attributed to the integration of three key concepts. Firstly, the incorporation of a TAM enables the synergy between spectral and spatial attention mechanisms. This collaboration better models the complex feature relationships in hyperspectral images, thereby enhancing the expressive capabilities of the classification model. Secondly, by appropriately setting dilation factors and incrementally expanding the network width in a pyramid-like structure, the method addresses the blind-spot issue of receptive fields typically found in standard dilated convolutions. This approach results in a larger receptive field, allowing the better differentiation of edge features for each class and, thus, producing clearer classification maps with reduced noise and speckles. Lastly, the use of cross-layer feature fusion techniques provides richer and more comprehensive feature information. This method not only introduces dilated convolutions and cross-layer connections within the RU blocks but also adds cross-layer connections between the overall DPRNs. This integration maintains the spatial resolution of the input and output while achieving a larger receptive field, enabling the network to capture more continuous and comprehensive contextual information.

#### 4.1.5. Convergence and Efficiency Analysis

**Figure 15:**
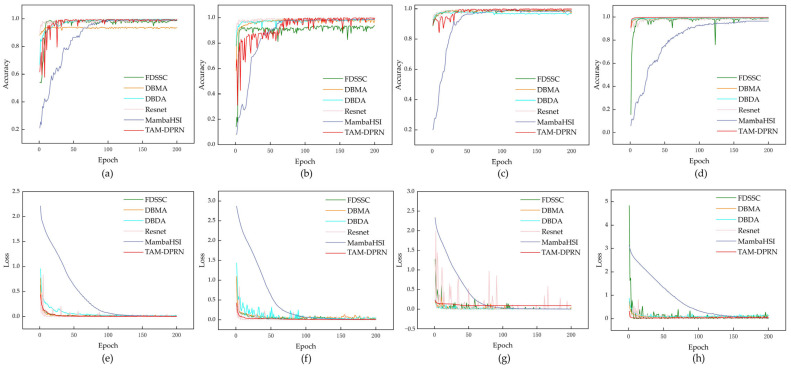
Variations in test accuracy versus training loss for various methods trained on different datasets. (**a**) Test accuracy on the UP dataset; (**b**) test accuracy on the SA dataset; (**c**) test accuracy on the TeaFarm dataset; (**d**) test accuracy on the HongHu dataset. (**e**) Training loss on the UP dataset; (**f**) training loss on the SA dataset; (**g**) training loss on the TeaFarm dataset; (**h**) training loss on the HongHu dataset.

This study further verifies the convergence of TAM-DPRN. Figure 15 illustrates the changes in loss values and accuracy over the course of iterations during the training process for the six different methods.

All methods show an upward trend in test accuracy as the number of training iterations increases across the four different datasets. This suggests that these methods can better generalize to unknown data in the later stages of training. The training losses decrease with each iteration and stabilize to some extent, indicating that each model gradually converges to a locally optimal solution during the training process.

The training loss of TAM-DPRN decreases rapidly in the early stages of training and stabilizes afterward, with no significant fluctuations during the subsequent training process. This indicates that the model can quickly and effectively learn specific spectral and spatial features that are useful for classification. Furthermore, the learned information is well-generalized to the test dataset, which is particularly evident in the SA and TeaFarm datasets. While TAM-DPRN may initially have slightly inferior performance compared with the other models, it quickly adapts and ultimately demonstrates outstanding performance. Notably, according to the loss curve, TAM-DPRN rapidly converges and stabilizes on the TeaFarm dataset after 25 epochs, showing no signs of overfitting. However, when dealing with the HongHu dataset, which has high dimensionality and a large data volume, coupled with imbalances in the class distribution, there may be slight loss fluctuations even after training stabilization. Nevertheless, in terms of test performance, TAM-DPRN’s classification accuracy surpasses that of other methods. Therefore, an analysis of the model’s performance in both the training and testing phases strongly suggests good convergence and generalization capabilities.

An excellent classification model should achieve not only high accuracy but also high efficiency. Table 11 presents a comparison of the average training and testing times per round for the five methods.

MambaHSI demonstrates exceptional computational efficiency across diverse hyperspectral datasets, particularly excelling in large-scale scenarios. MambaHSI employs dynamic spectral–spatial decoupling to separate the extraction of spatial features from the modeling of spectral sequences, thereby eliminating the redundant 3D convolutional operations that plague the DBMA and DBDA methods. For the TeaFarm dataset, TAM-DPRN exhibits slightly lower time efficiency than that of the FDSSC and DBMA methods, but it converges faster, demonstrating a more stable convergence trend after the 10th epoch. For the UP, SA, and Honghu datasets, the efficiency of TAM-DPRN is found to be second only to that of the MambaHSI method while demonstrating significantly faster processing speeds compared with the other methods. Especially for the large HongHu dataset, a notable improvement in efficiency is observed. In comparison with methods using 3D convolutional layers, TAM-DPRN, which is based on a 2D-CNN for spectral feature extraction, overcomes model redundancy, requiring fewer trainable parameters, and it reduces the time consumed during training and testing. It achieves higher accuracy and more stable performance.

### 4.2. Ablation Experiment and Analysis

To validate the impact of the TAM and the embedding of the TAM in RUs on network performance, a series of ablation experiments were conducted. Four different experimental conditions were designed, including using a TAM as the first layer of the model, embedding a TAM in an RU (RU-TAM), having only the spectral attention module in the embedded TAM (TAM-SE), and having only the spatial attention module in the embedded TAM (TAM-SA). Table 12 shows the overall accuracy metrics of the model with each module.

Compared with TAM-DPRN without the TAM as the first layer, there are noticeable improvements in OA and AA. Specifically, we observe improvements of 0.45% and 1.18%, 0.93% and 0.61%, 0.09% and 0.30%, and 0.59% and 1.71%, respectively. The Kappa coefficient also increases to different degrees. Therefore, before the data enter the CNN, the TAM is essential for improving the overall classification accuracy.

For the UP and TeaFarm datasets, which have fewer land-cover classes but a more dispersed distribution and smaller patches, the single-layer attention mechanism in the RU can further learn detailed features. By passing through the TAM, the network acquires most of the useful feature information, resulting in further improvements in accuracy. For the SA and HongHu datasets, the first layer of the TAM has already learned most of the features. As a result, in the RU, the attention mechanism only contributes slightly to the improvement in classification accuracy.

In summary, incorporating a TAM as the first layer of the model is crucial to a certain extent. This is because the tandem attention mechanism and the dense connectivity structure in the DPRM contribute to feature propagation, thereby enhancing classification performance. By embedding a TAM within each RU, the DPRM dynamically adjusts the importance of spectral and spatial features at multiple scales. This is more effective than methods such as DBDA, which apply attention mechanisms only at specific stages of the network. Overall, TAM-DPRN is more stable than the other methods, further emphasizing the rationality of the model architecture proposed in this study.

Furthermore, the rationale of the sequence of the spectral attention module and spatial attention module in the TAM is also validated. Table 13 presents the overall accuracy (OA) on various datasets with different sequences of attention modules.

The results indicate that, due to the high-dimensional spectral information in hyperspectral data, prioritizing processing spectral information allows the model to obtain more refined features at local pixel points. These detailed features can be integrated through the spatial attention mechanism, leading to better global feature fusion. In contrast, processing spatial information first may lead to feature mixing between different pixel points, thereby hindering the subsequent effectiveness of spectral attention.

**Figure 16:**
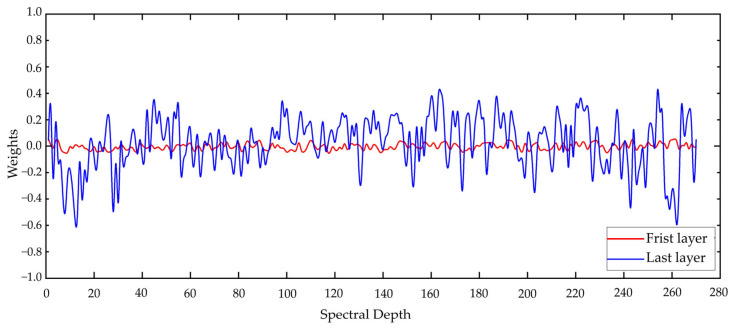
Weights generated by the spectrum-wise attention mechanism on the first layer and last layer on the HongHu dataset.

Subsequently, taking the HongHu dataset as an example, we examined the weights assigned to each spectral band by the spectral attention modules in the first and last layers of the network, as illustrated in Figure 16.

The figure reveals that the attention mechanism has a minimal impact on shallow features, with weights being concentrated near zero, indicating that these layers primarily perform preliminary screening and processing of the input data. As the layer depth increases, the attention mechanism becomes increasingly pivotal, guiding the network to prioritize the extraction of deep features, particularly focusing on the spectral bands that are most contributive to the classification task.

The attention mechanism in the final layer needs to comprehensively consider all of the feature information extracted from the preceding layers. These features are high-order characteristics resulting from multiple layers of nonlinear transformations. Since these high-order features have integrated multi-level spectral information, the network may assign greater weights to certain critical bands to further distinguish different classes. Consequently, the attention mechanism in the final layer assigns higher weights to the most contributive bands to maximize the overall classification performance.

Overall, extracting spectral features first enables the model to better adapt to the high-dimensional characteristics of hyperspectral data, reducing instability factors such as noise during training. Therefore, the sequence of spectral attention followed by spatial attention is more consistent with the design logic of the model.

## 5. Conclusions

This study proposes a method called TAM-DPRN, which combines a tandem spectral–spatial attention mechanism with a dense pyramidal residual network for hyperspectral image classification. The model is an end-to-end, on-the-fly training and validation framework based on a CNN. The first layer of the model is a concatenated attention module (TAM), which allows the model to adaptively identify spectral bands and informative pixels that are useful for HSIC. The basic building blocks of the CNN are RUs, which are used to learn the detailed features of the data. The dense connections between RUs enable the utilization of multiple scales at each layer, using all feature maps from the previous layer to compute the feature maps for each layer. This captures more comprehensive spectral and spatial information.

Notably, TAM-DPRN employs 2D convolutional kernels instead of 3D convolutional kernels to enhance useful spectral information while diminishing redundancy through the spectral attention mechanism, which provides higher accuracy and more stable performance. Additionally, the final RU uses dilated convolutions with a reasonable dilation rate instead of traditional convolutions to learn features at different scales. Experiments on four public datasets demonstrate the strong classification performance of TAM-DPRN for different types of data and that it has good generalization.

Our model was composed of only 84 layers, which made the network relatively shallow. During the experiment, each module was subjected to a heavy load. Although better results were achieved, the time efficiency could be further improved compared with MambaHSI. More effectively selecting training samples to train the network and enhancing the computational efficiency of large-scale datasets are the first tasks for future research. In addition, with the development of hyperspectral image sensors, the scope has been expanded beyond the publicly available datasets commonly used by the public. Therefore, the second task for future research is to attempt to introduce ultra-low-resolution datasets, such as Hyperion data, to evaluate the model’s tolerance to the decline in data quality and to explore cross-modal transfer learning, such as joint visible–hyperspectral learning, to enhance cross-sensor capabilities.

## Figures and Tables

**Table 1:** Ground object class and sample numbers in the UP dataset.

	Class	Name	Legend	Samples
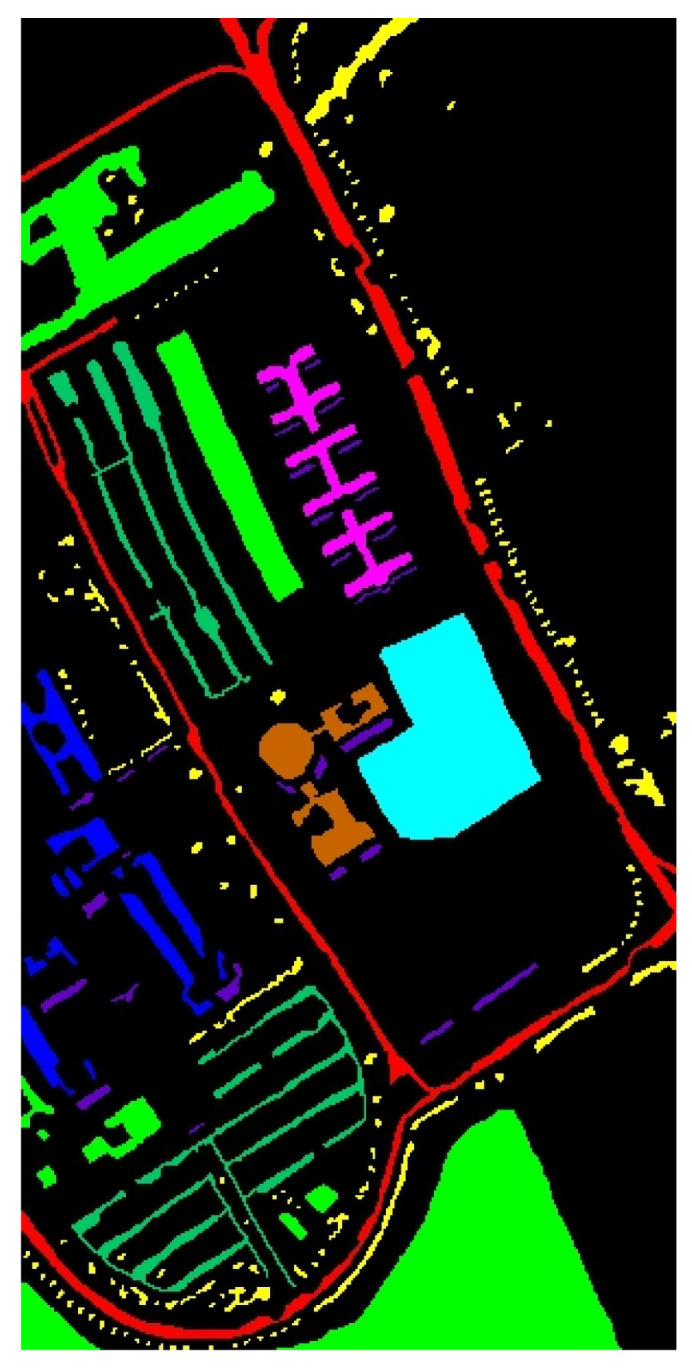	1	Asphalt		6631
	2	Meadows		18,649
	3	Gravel		2099
	4	Trees		3064
	5	Painted Metal Sheets		1345
	6	Bare Soil		5029
	7	Bitumen		1330
	8	Self-Blocking Bricks		3682
	9	Shadows		947

**Table 2:** Ground object class and sample numbers in the SA dataset.

	Class	Name	Legend	Samples
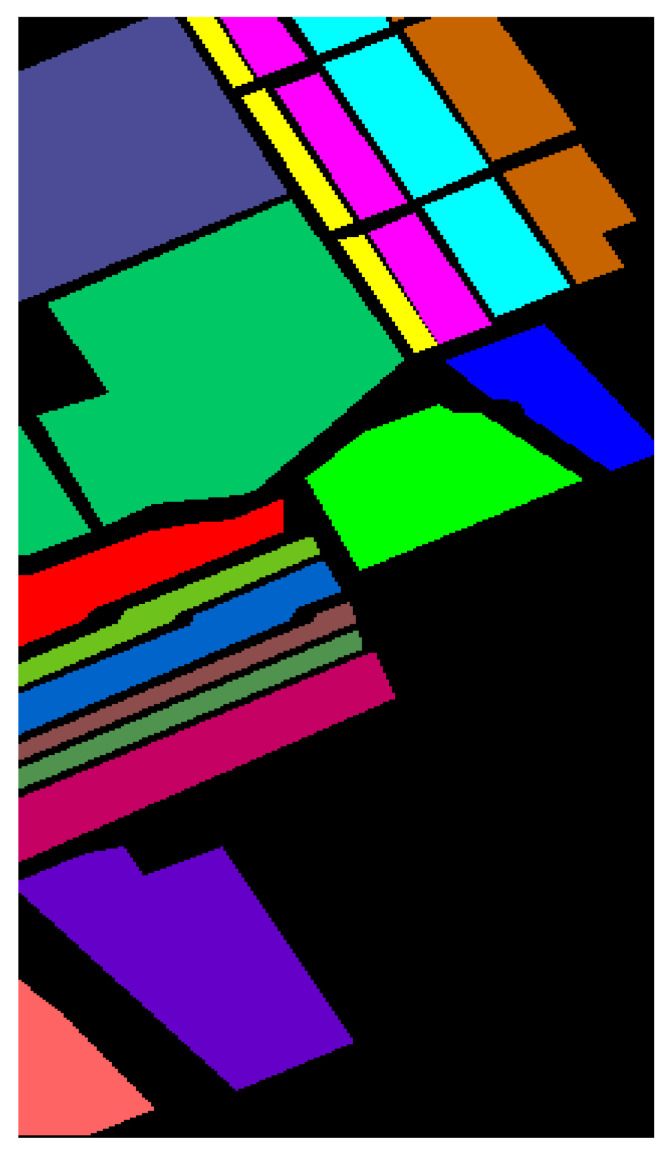	1	Brocoli_green_weeds_1		2009
	2	Brocoli_green_weeds_2		3726
	3	Fallow		1976
	4	Fallow_rough_plow		1394
	5	Fallow_smooth		2678
	6	Stubble		3959
	7	Celery		3579
	8	Grapes_untrained		11,271
	9	Soil_vinyard_develop		6203
	10	Corn_senesced_green_weeds		3278
	11	Lettuce_romaine_4wk		1068
	12	Lettuce_romaine_5wk		1927
	13	Lettuce_romaine_6wk		916
	14	Lettuce_romaine_7wk		1070
	15	Vinyard_untrained		7268
	16	Vinyard_vertical_trellis		1807

**Table 3:** Ground object class and sample numbers in the TeaFarm dataset.

	Class	Name	Legend	Samples
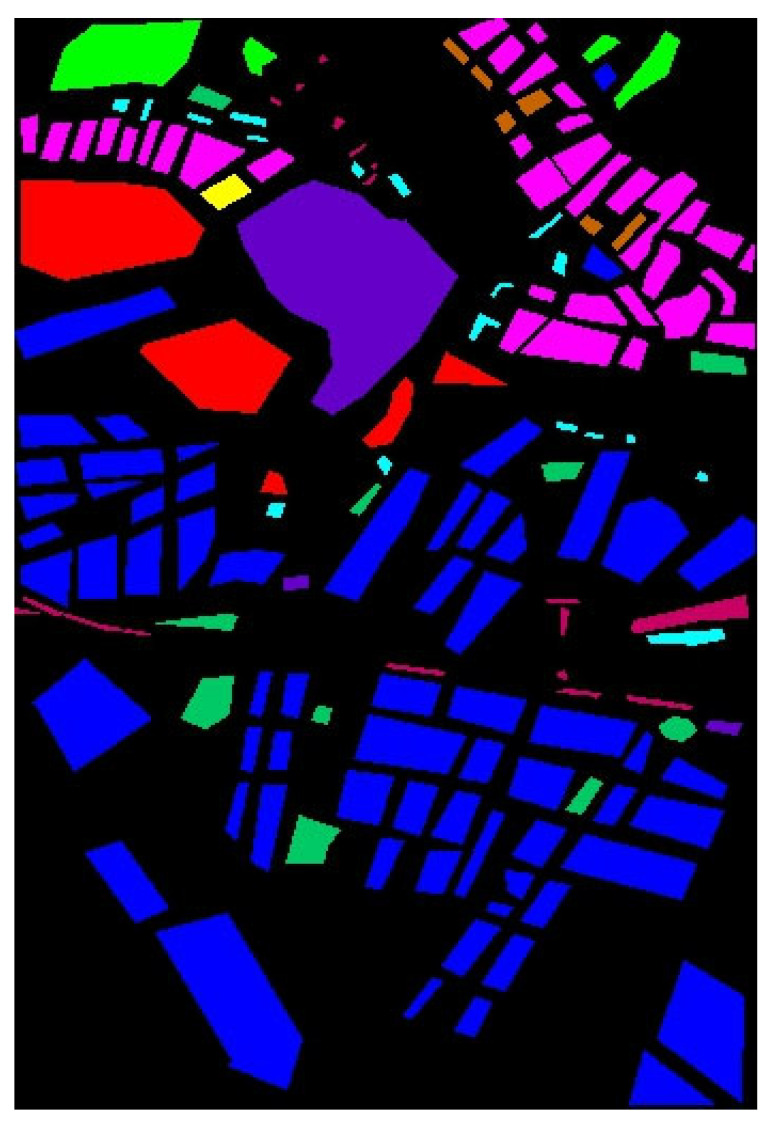	1	Masson Pine		5806
	2	Bamboo forest		2318
	3	Tea plant		28,428
	4	Reed		214
	5	Rice paddy		6809
	6	Sweet potato		817
	7	Caraway		429
	8	Weed		1861
	9	Water		6141
	10	Building/road		911

**Table 4:** Hyperparameter settings for each dataset.

Datasets	Hyperparameters			
	Dataset Partitioning	Learning Rate	Spatial Input Size	Batch Size
UP	1.5:1:7.5	0.0002	13 × 13	11
SA	3:1:6	0.0001	17 × 17	16
TeaFarm	2:1:7	0.0001	13 × 13	11
HongHu	3:1:6	0.0001	15 × 15	13

**Table 5:** Classification results on the four datasets with different partitioning ratios.

Dataset	Classification Metrics	Partition Ratios						
		1:1:8	1.5:1:7.5	2:1:7	2.5:1:6.5	3:1:6	3.5:1:5.5	4:1:5
UP	OA	99.33	99.60	99.54	99.15	99.26	99.26	99.35
	AA	98.85	99.49	99.29	98.68	98.83	98.92	98.95
	Kappa × 100	99.11	99.48	99.39	98.88	99.02	99.02	99.13
SA	OA	99.83	99.84	99.87	99.78	99.95	99.87	99.79
	AA	99.80	99.89	99.90	99.74	99.94	99.85	99.76
	Kappa × 100	99.81	99.82	99.85	99.76	99.95	99.85	99.76
TeaFarm	OA	99.60	99.63	99.81	99.77	99.68	99.75	99.54
	AA	98.87	98.69	99.40	98.93	98.82	99.32	98.07
	Kappa × 100	99.41	99.46	99.72	99.66	99.53	99.64	99.32
HongHu	OA	99.47	99.35	99.46	99.55	99.83	99.61	99.57
	AA	98.42	98.19	98.53	98.90	99.56	98.84	98.84
	Kappa × 100	99.33	99.18	99.31	99.43	99.78	99.50	99.46

**Table 6:** Classification results on the four datasets with different optimizers.

	UP	SA	TeaFarm	HongHu
Adam	96.46	99.89	98.81	99.18
AdamW	99.23	99.90	99.78	99.74
Nadam	99.44	99.87	99.26	98.78
RMSprop	99.60	99.95	99.81	99.84

**Table 7:** Classification results on the UP dataset.

Class	SVM	FDSSC	DBMA	DBDA	ResNet34	MambaHSI	TAM-DPRN
1	91.97	98.74	83.84	99.94	99.27	92.94	99.14
2	85.10	99.40	98.36	99.96	98.84	93.57	99.90
3	71.15	99.79	98.76	98.50	98.45	96.84	98.82
4	95.38	98.45	98.01	90.53	99.71	90.61	99.44
5	99.55	99.47	99.63	97.42	99.90	100.00	100.00
6	88.07	99.02	94.07	100.00	98.53	99.28	99.86
7	80.39	100.00	100.00	99.26	98.32	97.67	99.13
8	75.35	95.21	77.51	95.03	90.64	95.33	99.11
9	99.78	99.89	99.66	98.32	100.00	99.67	100
OA	84.08	98.86	93.03	98.56	98.21	94.70	99.60
AA	87.42	98.89	94.43	97.66	98.18	96.21	99.49
Kappa × 100	79.84	98.49	90.88	98.09	97.61	98.75	99.48

**Table 8:** Classification results on the SA dataset.

Class	SVM	FDSSC	DBMA	DBDA	ResNet34	MambaHSI	TAM-DPRN
1	99.89	99.60	100.00	100.00	100.00	100.00	100.00
2	99.42	99.96	99.92	100.00	100.00	100.00	100.00
3	95.58	97.44	100.00	98.89	99.98	99.85	99.86
4	99.42	97.36	95.42	93.58	99.19	100.00	100.00
5	98.15	99.10	99.03	99.60	99.77	97.01	99.95
6	99.65	99.72	99.74	100.00	100.00	100.00	100.00
7	97.38	99.02	99.97	98.22	100.00	99.94	100.00
8	76.05	88.92	98.28	99.65	98.33	95.37	99.99
9	97.72	99.53	99.85	97.24	99.97	99.87	99.77
10	89.94	95.70	97.71	97.85	99.91	99.51	99.69
11	92.00	94.89	100.00	90.75	99.39	100.00	100.00
12	97.82	96.70	99.58	100.00	100.00	100.00	100.00
13	97.22	99.09	98.46	100.00	99.54	100.00	100.00
14	97.94	96.70	99.21	96.18	99.52	99.90	100.00
15	72.94	89.16	83.62	81.42	93.50	96.94	99.98
16	97.65	99.45	100.00	100.00	99.95	100.00	100.00
OA	89.78	95.04	96.70	95.81	98.62	98.41	99.95
AA	94.30	97.02	98.17	97.09	99.32	99.27	99.94
Kappa × 100	88.56	94.46	96.32	95.32	98.47	98.67	**99.95**

**Table 9:** Classification results on the TeaFarm dataset.

Class	SVM	FDSSC	DBMA	DBDA	ResNet34	MambaHSI	TAM-DPRN
1	73.51	99.73	99.09	98.99	99.13	99.90	99.98
2	19.76	95.34	99.70	98.42	98.68	95.00	98.58
3	94.48	99.30	98.47	99.86	99.06	93.36	99.97
4	66.18	100.00	100.00	100.00	99.75	100.00	100
5	94.73	100.00	99.97	100.00	99.98	99.99	100
6	81.69	100.00	99.70	95.06	99.86	98.97	99.82
7	91.22	100.00	100.00	100.00	97.65	100.00	99.33
8	42.43	92.94	98.45	98.93	96.02	97.86	98.16
9	98.93	100.00	100.00	100.00	100	99.79	99.93
10	51.48	100.00	100.00	90.26	100	100.00	98.27
OA	84.84	99.13	99.01	99.14	99.19	96.12	99.81
AA	71.44	98.73	99.54	98.15	99.01	98.48	99.40
Kappa × 100	74.62	98.71	98.53	98.72	98.81	100.00	99.72

**Table 10:** Classification results on the HongHu dataset.

Class	SVM	FDSSC	DBMA	DBDA	ResNet34	MambaHSI	TAM-DPRN
1	94.75	99.91	99.17	98.61	99.33	95.68	99.22
2	77.29	93.91	99.26	96.05	97.19	97.64	99.78
3	82.23	96.91	96.90	98.80	98.18	94.73	99.67
4	92.11	99.95	99.80	100.00	99.28	95.55	99.98
5	58.93	95.90	99.04	100.00	94.56	98.67	99.84
6	85.95	99.85	99.11	100.00	99.08	95.31	100.00
7	68.95	98.17	97.83	97.91	95.52	87.52	99.15
8	70.82	95.14	97.42	97.94	95.62	95.96	99.79
9	97.38	99.31	99.62	98.91	99.88	96.62	100.00
10	64.70	98.59	98.82	98.95	98.06	88.16	99.84
11	61.04	97.58	99.40	99.71	93.65	86.85	99.71
12	69.52	93.87	99.91	99.85	98.30	93.43	99.86
13	67.59	96.61	98.01	99.70	95.88	90.84	99.73
14	89.87	98.77	97.43	99.58	99.26	95.24	99.88
15	85.14	98.96	99.74	98.78	97.83	100.00	99.28
16	87.06	99.54	99.67	99.93	99.74	99.16	99.98
17	84.62	93.18	97.11	99.67	92.78	99.76	100.00
18	56.69	98.56	99.10	98.70	95.19	95.94	99.87
19	75.97	99.37	98.76	98.95	96.42	89.59	99.93
20	79.37	95.24	98.68	98.61	99.03	98.37	99.84
21	0.00	88.15	98.95	100.00	86.64	100.00	99.89
22	60.81	96.95	98.90	99.97	98.95	98.55	99.93
OA	84.61	98.79	99.09	98.84	98.21	94.32	99.84
AA	73.22	97.02	98.76	99.12	96.84	95.16	99.78
Kappa × 100	78.90	98.46	98.85	98.53	97.74	96.19	99.80

**Table 11:** The average training time and testing time of the different methods on different datasets in each epoch.

Dataset	Time	FDSSC	DBMA	DBDA	ResNet34	MambaHSI	TAM-DPRN
UP	Training time (s)	8.10	4.46	13.73	9.34	3.22	4.84
	Testing time (s)	0.50	0.60	1.85	0.27	0.17	0.53
SA	Training time (s)	15.60	7.26	24.10	31.28	1.76	6.30
	Testing time (s)	1.30	1.60	4.80	0.49	0.15	0.47
TeaFarm	Training time (s)	3.10	1.96	6.04	24.88	1.71	6.10
	Testing time (s)	0.55	0.65	1.85	0.39	0.21	0.72
HongHu	Training time (s)	389	161	573	120	2.39	53
	Testing time (s)	1.45	1.65	4.67	2.10	0.22	1.28

**Table 12:** Results of the ablation experiments.

TAM	RU- TAM	TAM- SE	TAM- SA	UP			SA			TeaFarm			HongHu		
				OA%	AA%	K × 100	OA%	AA%	K × 100	OA%	AA%	K × 100	OA%	AA%	K × 100
	√	√	√	99.15	98.31	98.87	99.02	99.31	98.91	99.72	99.10	99.59	99.25	98.61	99.06
√				99.21	98.81	98.95	99.87	99.87	99.86	99.70	99.22	99.65	99.57	98.93	99.46
√	√	√		99.40	98.98	99.21	99.89	99.87	99.82	99.72	98.86	99.59	99.64	98.97	99.55
√	√		√	99.42	99.04	99.23	99.84	99.89	99.82	99.76	99.07	99.65	99.59	98.86	99.49
√	√	√	√	99.60	99.49	99.48	99.95	99.94	99.95	99.81	99.40	99.72	99.84	99.78	99.80

**Table 13:** OA of TAM-DPRN with different sequences of attention modules.

Attention Modules	UP	SA	TeaFarm	HongHu
SA+SE	99.14%	99.73%	99.77%	99.71%
SE+SA	99.60%	99.95%	99.81%	99.84%

## Data Availability

The four public datasets used in this study can be found and experimented upon at https://www.ehu.eus/ccwintco/index.php?title=Hyperspectral_Remote_Sensing_Scenes#Salinas_scene, https://doi.org/10.3974/geodb.2017.03.04.V1, http://rsidea.whu.edu.cn/resource_WHUHi_sharing.htm (accessed on 13 September 2023).
